# Association of host protein VARICOSE with HCPro within a multiprotein complex is crucial for RNA silencing suppression, translation, encapsidation and systemic spread of potato virus A infection

**DOI:** 10.1371/journal.ppat.1008956

**Published:** 2020-10-12

**Authors:** Swarnalok De, Maija Pollari, Markku Varjosalo, Kristiina Mäkinen

**Affiliations:** 1 University of Helsinki, Department of Microbiology and Viikki Plant Science Centre, Finland; 2 Institute of Biotechnology, University of Helsinki, Finland; University of Cambridge, UNITED STATES

## Abstract

In this study, we investigated the significance of a conserved five-amino acid motif ‘AELPR’ in the C-terminal region of helper component–proteinase (HCPro) for potato virus A (PVA; genus *Potyvirus*) infection. This motif is a putative interaction site for WD40 domain-containing proteins, including VARICOSE (VCS). We abolished the interaction site in HCPro by replacing glutamic acid (E) and arginine (R) with alanines (A) to generate HCPro^WD^. These mutations partially eliminated HCPro-VCS co-localization in cells. We have earlier described potyvirus-induced RNA granules (PGs) in which HCPro and VCS co-localize and proposed that they have a role in RNA silencing suppression. We now demonstrate that the ability of HCPro^WD^ to induce PGs, introduce VCS into PGs, and suppress RNA silencing was impaired. Accordingly, PVA carrying HCPro^WD^ (PVA^WD^) infected *Nicotiana benthamiana* less efficiently than wild-type PVA (PVA^WT^) and HCPro^WD^ complemented the lack of HCPro in PVA gene expression only partially. HCPro was purified from PVA-infected leaves as part of high molecular weight (HMW) ribonucleoprotein (RNP) complexes. These complexes were more stable when associated with wild-type HCPro than with HCPro^WD^. Moreover, VCS and two viral components of the HMW-complexes, viral protein genome-linked and cylindrical inclusion protein were specifically decreased in HCPro^WD^-containing HMW-complexes. A VPg-mediated boost in translation of replication-deficient PVA (PVA^ΔGDD^) was observed only if viral RNA expressed wild-type HCPro. The role of VCS-VPg-HCPro coordination in PVA translation was further supported by results from VCS silencing and overexpression experiments and by significantly elevated PVA-derived *Renilla* luciferase vs PVA RNA ratio upon VPg-VCS co-expression. Finally, we found that PVA^WD^ was unable to form virus particles or to spread systemically in the infected plant. We highlight the role of HCPro-VCS containing multiprotein assemblies associated with PVA RNA in protecting it from degradation, ensuring efficient translation, formation of stable virions and establishment of systemic infection.

## Author summary

This study revealed that the potyviral helper component proteinase (HCPro) and the host protein VARICOSE (VCS) are linked in a manner that is important for suppression of RNA silencing, formation of potyvirus-induced RNA granules, translation of viral proteins, stability of virions, and development of systemic potato virus A (PVA) infection. The results suggest that HCPro and VCS belong to the core components of large RNP complexes regulating PVA infection. We suggest that these complexes protect viral RNAs in the cytoplasm after release from the replication complex and direct them to translation and intact to the viral particles.

## Introduction

The host-virus relationship is a quintessential example of the evolutionary ‘arms race’. The most intriguing aspect of this molecular struggle is that while the hosts have large genomes with a huge range of defences, viral genomes in comparison are minuscule. Yet they successfully manage to hijack host cellular machineries to serve their propagation. In order to cope with the constant selection pressure and to include all the essential genetic information within a tiny genome, viruses encode proteins which are multifunctional in nature.

For example potyviral helper component-proteinase (HCPro) is involved in interactions with multiple host proteins. It carries out many essential functions during the viral infection cycle. Albeit best known for its role as an RNA silencing suppressor, HCPro’s primary functions include polyprotein processing [1,2] and viral plant-to-plant transmission [3]. Moreover, it is involved in viral genome replication and movement [4–7], particle encapsidation [8], interactions with proteasome [9], the formation of RNA granules (PGs) and translational regulation [10]. In recent years, many independent studies have established HCPro as an interacting partner for a wide array of host factors involved in different cellular pathways. These include but are not limited to S-adenosyl methionine synthetase (SAMS) and S-adenosyl homocysteine hydrolyse (SAHH) [11,12], eukaryotic initiation factor 4E and (iso)4E (eIF4E/(iso)4E) [13], microtubule-associated proteins HIP1[14] and HIP2 [15], ARGONAUTE 1 (AGO1) [12] and a calmodulin-like protein rgs-CaM [16]. Many HCPro-induced transcriptional changes in infected plants occur via the RAV2 transcription factor [17]. Furthermore, HCPro interacts with ribosomes in PVA-infected plants [12,18]. In Ivanov et al. [12], we suggested that polysome-associated high molecular weight complexes including HCPro and ARGONAUTE 1 (AGO1) may function in translational regulation [12,18].

Here we show that the C-terminal region of HCPro contains a highly conserved five amino acid motif. This motif has the potential to interact with WD40 domain proteins, a large family of eukaryotic proteins [19,20]. The defining feature of this protein family is the presence of WD40 domains, which are tandemly repeated stretches of approximately 40–60 amino acids beginning with Gly-His (GH) and ending with Trp-Asp (WD) [20,21]. WD40 repeat domains commonly fold into a multiple beta-propeller structure with an overall doughnut or open-ended cylinder shape (reviewed by [22,23]; also see S1 Fig). These domains provide a platform for the reversible assembly of multiprotein complexes [24,25]. WD40 domain proteins serve often as hubs of cellular networks and are therefore key players in many biological processes [21,22,26]. In Hafrén et al. [10], we proposed that potyvirus-induced RNA granules (PGs) are structures involved in the protection of viral RNAs (vRNAs) against the host’s RNA silencing machinery. A WD40 domain protein VARICOSE (VCS) localizes in PGs [10]. In host cells VCS is involved in the assembly of protein complexes associated with RNA metabolism and it is crucial for seedling development [27]. In the context of PVA infection VCS is an important infectivity factor [10]. In this study, we designed an HCPro mutant in which a potential interaction site for WD40 domain proteins was abolished. Interestingly, this mutation impaired HCPro-VCS co-localization *in planta* and negatively affected the assembly and stability of HCPro-VCS-associated multiprotein complexes. Furthermore, the virus carrying this mutation demonstrated major defects in multiple stages of infection. Cumulatively he results advocate the biological significance of the HCPro-VCS interaction in the PVA infection cycle. In addition, this study illuminates the formation and function of large RNA-protein assemblies in the regulation of infection.

## Results

### Identification of a conserved binding site for WD40-domain proteins in PVA HCPro

**Fig 1:**
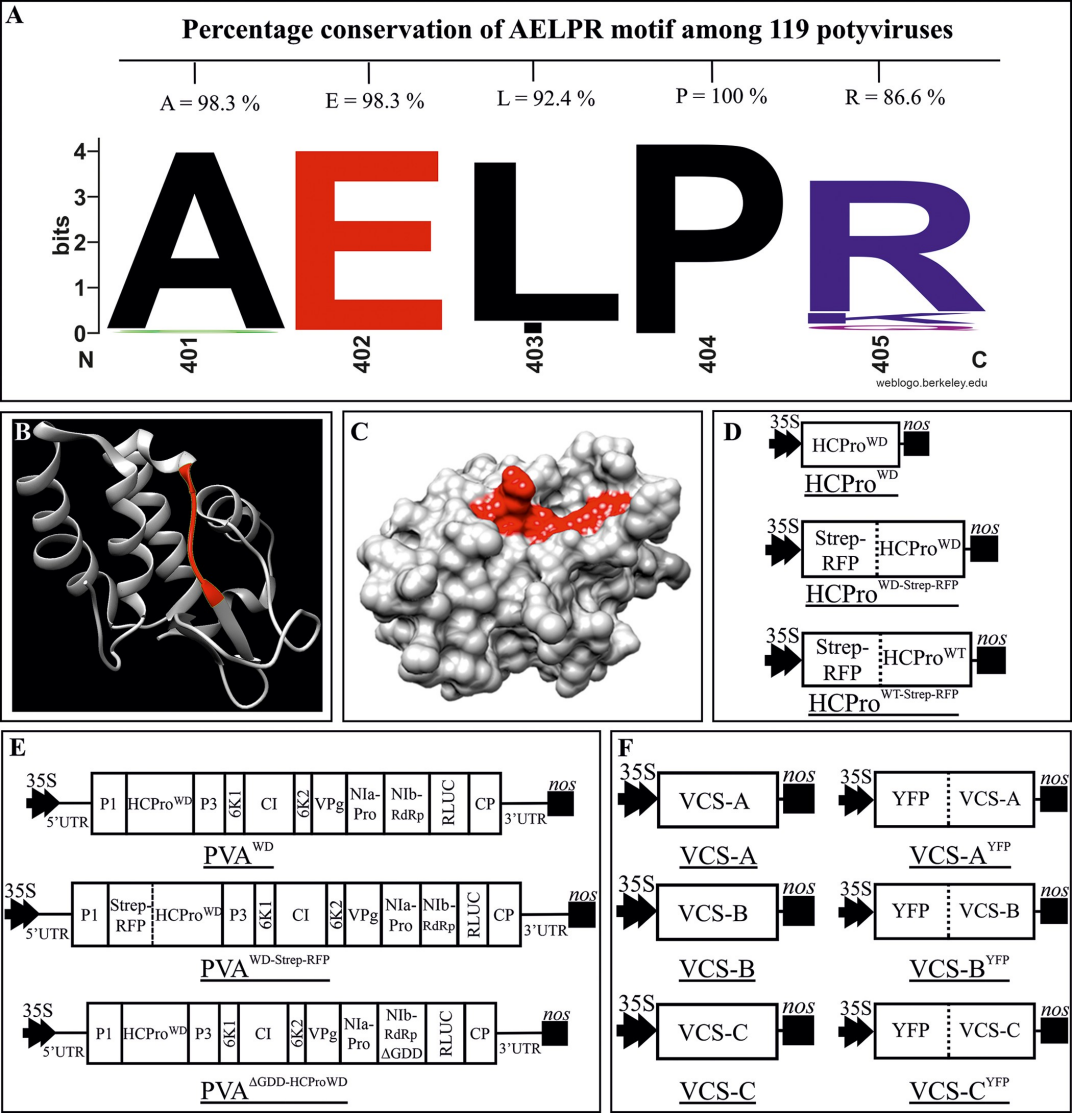
AELRP sequence in HCPro is predicted to bind to proteins containing a WD40-domain. (A) Percentage of amino acid conservation within the AELPR motif in 119 potyviral HCPro sequences. This motif was identified by scanning the PVA HCPro sequence for short linear motifs (SLiMs) via ELM server (http://elm.eu.org/). The AELPR motif was predicted to be a putative WD40 -domain-interacting motif. (B) Ribbon diagram of X-ray crystallographic structure of the cysteine protease domain of TuMV HCPro (PDB ID: 3RNV). Secondary structure of the ‘AELPR’ motif has been highlighted in red. (C) Surface diagram of 3RNV showing the accessibility of the WD40-domain -interacting motif in HCPro (highlighted in red). (D—F) Schematic representation of the constructs prepared for this study (not in scale): (D) HCPro overexpression constructs. (E) icDNAs of the full length PVA constructs. (F) VCS overexpression constructs.

The C-terminal region of PVA HCPro contains a five amino acid motif ‘AELPR’. This sequence is highly conserved in the genus *Potyvirus* (Fig 1A and S1A Fig). The percentage of conservation of the individual residues in the AELPR motif ranges from 86.6 to 100% in the HCPro sequences of the 119 potyvirus species we compared. Alanine, glutamic acid and proline were the most conserved residues (Fig 1A). This motif falls within the known crystal structure of the C-terminal cysteine proteinase domain of HCPro of turnip mosaic virus (TuMV; genus *Potyvirus*) [28] (Fig 1B). The catalytically active residues surround the motif as visible from the sequence comparison presented in (S1A Fig; marked by arrows). It is an inherently linear region between an α-coil and a β-sheet (highlighted in red) on the HCPro surface (Fig 1C) thus enabling direct interactions with possible binding partners. Based on predictions (with P < 0.05) by the Eukaryotic Linear Motif (ELM) Resource for Functional Sites in Proteins (http://elm.eu.org/), there are three motifs overlapping with ‘AELPR’ between amino acids 398–406 ‘TSA**AEL**’, ‘SA**AELPR**I’ and ‘**ELPR**I’, which have the potential to act as a short linear motif capable of interacting with WD40 domain-containing proteins. In the stretch 5 in the alignment of S1A Fig at least one of these motifs is retained leading to 100% conservation in the 119 potyvirus sequences. A molecular docking model (created using ClusPro online server: https://cluspro.bu.edu/) between TuMV HCPro and VCS suggested the presence of the ‘AELPR’ motif at the site of interaction (S1B–S1F Fig). Because of the properties described above, we hypothesized that this sequence could be the binding site to host proteins containing WD40 domains. The AELPR motif was mutated by replacing the charged residues glutamic acid and arginine with alanines. These mutations disrupted all the three possible forms, ‘TSA**AEL**’, ‘SA**AELPR**I’ and ‘**ELPR**I’, of WD40 domain- interacting motifs. The mutated virus and protein were named PVA^WD^ and HCPro^WD^, respectively. Fig 1D and 1E depicts the PVA and HCPro constructs that were prepared for this study. All constructs used in this study are described in the S1 Table (see also S1 Reference).

### Infection profile of PVA^WD^

**Fig 2:**
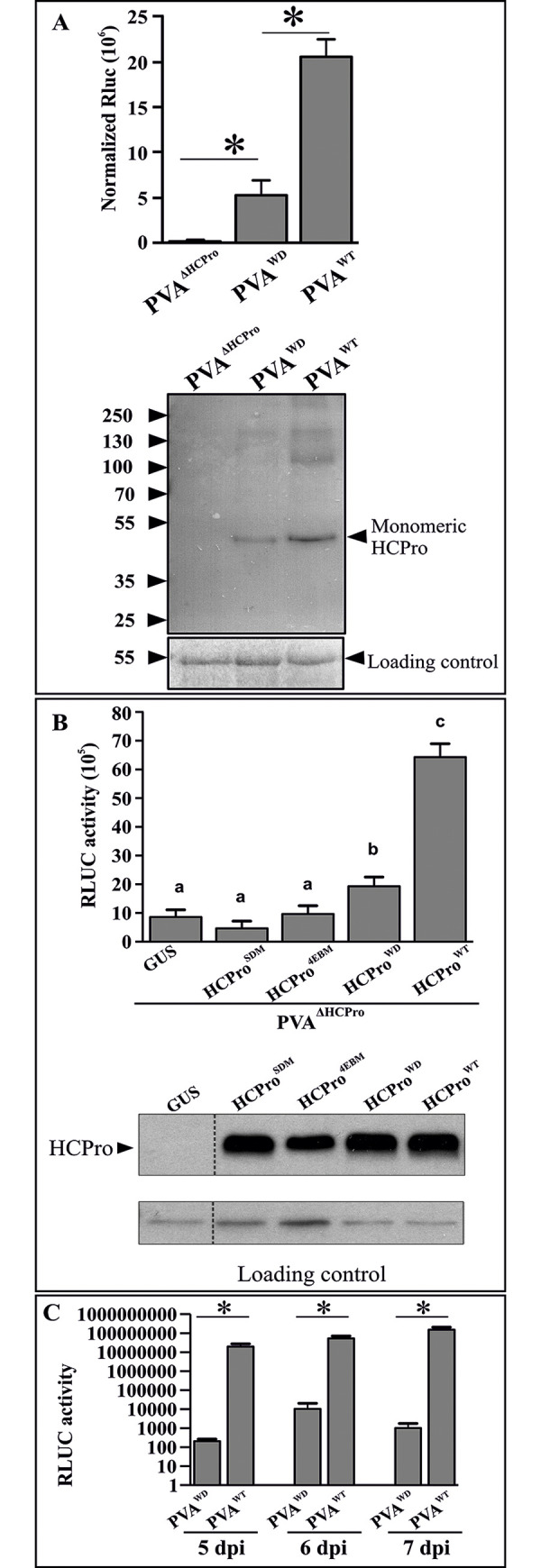
HCPro^WD^ mutation reduces local and prevents systemic PVA infection without affecting its autocatalytic proteinase activity. (A) Virus-derived RLUC activity levels of PVA^WT^ and PVA^WD^ to evaluate the effect of the mutation in WD40-domain -interacting motif of HCPro on PVA gene expression. PVA^WD^ exhibits a ~5 fold lower expression level than PVA^WT^. All the constructs were infiltrated at OD_600_ = 0.01, and the samples were collected at 5 dpi. A western blot analysis with α-HCPro antibody of the same samples is presented in the lower panel. The presence of monomeric HCPro^WD^ derived from PVA^WD^ confirms the preservation of the autocatalytic activity of HCPro^WD^’s cysteine ​​protease domain. Statistically significant difference between the samples is denoted by an asterisk (*P < 0.05; n = 3). (B) Complementation of PVA^ΔHCPro^ infectivity by various HCPro mutants. *Agrobacterium* carrying different HCPro variants were infiltrated as follows: silencing-deficient HCPro^SDM^ and eIF4E binding-deficient HCPro^4EBM^ mutants at OD_600_ = 1, HCPro^WD^ and HCPro^WT^ at OD_600_ = 0.3. PVA^ΔHCPro^ was infiltrated at OD_600_ = 0.1. GUS was used as the control, as well as to equalize infiltrated *Agrobacterium* cell numbers among all the sets. Firefly luciferase (FLUC) at OD_600_ = 0.01 was added as internal control for normalization of RLUC activity. Samples were collected at 3 dpi. A western blot analysis (lower panel) with α-HCPro antibodies demonstrates comparative expression levels of all HCPro variants from their corresponding expression constructs. Different letters above the bars indicate a statistically significant difference (student’s t-test *P* < 0.05; n = 4). (C) A time course experiment of systemic infection by PVA^WD^. *N*. *benthamiana* leaves were infiltrated with *Agrobacterium* carrying PVA^WD^ / PVA^WT^ constructs (OD_600_ = 0.01). Samples were collected from newly emerging systemic leaves at 5, 6, 7 dpi, respectively and RLUC activity was measured. Statistical significance was assessed using student’s *t*-test (*P < 0.05; n = 3).

In order to investigate the effects of the mutation in HCPro^WD^ on PVA infection we measured viral expression levels from local and systemic leaves. Expression of the viral polyprotein was assessed from the activity levels of the RLUC reporter gene incorporated between the NIb and CP cistrons [29]. In local leaves, PVA^WD^ accumulated with approximately five-fold lower efficiency than PVA^WT^ (Fig 2A). As the mutated motif lies in close vicinity to the cysteine protease domain of HCPro, it was necessary to confirm that this mutation did not hamper its autocatalytic activity. A western blot analysis of the same samples was carried out with an α-HCPro antibody (lower panel Fig 2A). HCPro^WD^ accumulation level was lower than that of wild-type HCPro (HCPro^WT^) in the blot, which was in line with the difference in the polyprotein expression levels of PVA^WD^ and PVA^WT^. The presence of monomeric HCPro^WD^ derived from PVA^WD^ infection (Fig 2A) confirmed that HCPro^WD^ had retained its polyprotein processing ability.

Additionally, we compared the complementation efficiency of HCPro^WD^ with respect to HCPro^WT^ and two other HCPro mutants, the RNA silencing suppression-deficient HCPro (HCPro^SDM^) and eIF(iso)4E binding-deficient HCPro (HCPro^4EBM^) [10]. For this, we infiltrated the plants with PVA^ΔHCPro^ and complemented the infection with GUS (OD_600_ = 1), HCPro^SDM^ (OD_600_ = 1), HCPro^4EBM^ (OD_600_ = 1), HCPro^WD^ (OD_600_ = 0.3), or HCPro^WT^ (OD_600_ = 0.3). Neither HCPro^SDM^ nor HCPro^4EBM^ could elevate RLUC expression from PVA^ΔHCPro^ showing no sign of complementation. HCPro^WD^ on the other hand promoted PVA^ΔHCPro^ gene expression significantly (Fig 2B) and HCPro^WT^ complemented PVA^ΔHCPro^ most efficiently. Interestingly, the complementation efficiencies of HCPro^WD^ and HCPro^WT^ also exhibited approximately a five-fold difference, which was similar to differences in the gene expression efficiency of PVA^WD^ and PVA^WT^ (Fig 2A). Infiltration ODs of all the HCPros were adjusted to achieve equal expression (lower panel of Fig 2B). Notably, same infiltration ODs of HCPro^WD/WT^ resulted in similar level of HCPro expression (lower panel of Fig 2B; also see S2 Fig for the expression levels of their Strep-RFP tagged versions). Therefore, the defective infection in PVA^WD^ is not because of the lower expression level of HCPro^WD^, rather it is something related to its functional aspects.

An interesting difference in the viral gene expression was noticed while monitoring the infection level of PVA^WD^ and PVA^WT^ in systemically infected leaves. There was more than a 1000-fold reduction in the RLUC activity in PVA^WD^ compared to PVA^WT^ (Fig 2C) indicating that the systemic spread of PVA^WD^ was severely compromised. Despite the difference, the low level of PVA^WD^ expression was detectable in the systemic leaves already at 5 dpi. We followed the development of RLUC activity at 6 and 7 dpi, but did not see any signs that the mutant would reach wild-type expression levels. Since HCPro can affect the potyviral infection cycle at multiple levels we next assessed the effect of the HCPro^WD^ mutation on further known properties of HCPro.

### The silencing suppression capacity of the HCPro^WD^ mutant is compromised

**Fig 3:**
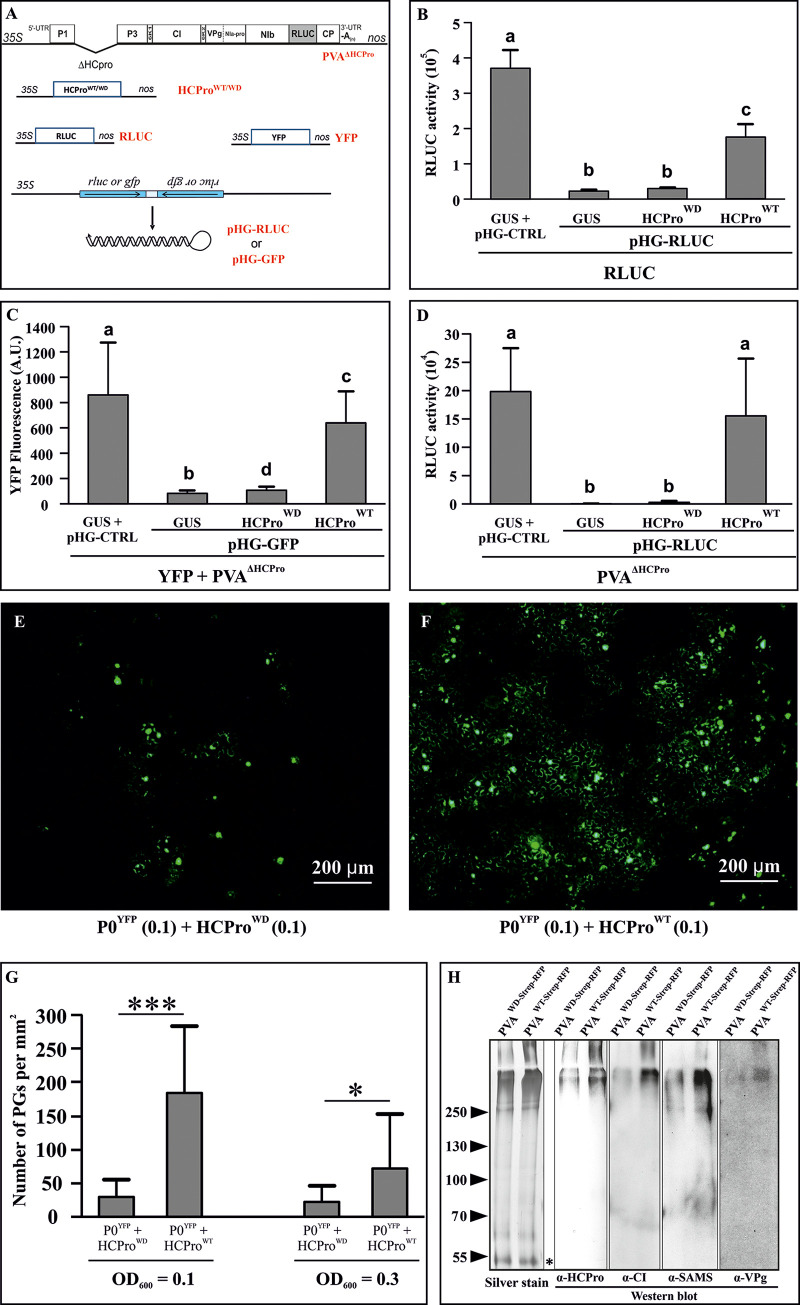
HCPro^WD^ is defective in suppressing hairpin-triggered RNA silencing, inducing PGs and forming infection associated HMW complexes. (A) A schematic representation of the constructs used to study the suppression of hairpin-triggered RNA silencing. The hairpin RNAs (pHG-RLUC and pHG-GFP) targeting monocistronic RLUC, YFP, and RLUC-expressing PVA RNA were expressed to trigger RNA silencing. HCPro^WD^ and HCPro^WT^ were expressed to test their capacity to suppress RNA silencing. (B) The capacity of HCPro^WD^ to suppress RNA silencing. *N*. *benthamiana* plants were agroinfiltrated with the RLUC expression construct (OD_600_ = 0.01), pHG-RLUC construct (OD_600_ = 0.4) and HCPro^WD^ / HCPro^WT^ constructs (OD_600_ = 0.3) as indicated. Empty pHG-CTRL and GUS were expressed as the controls. All constructs were co-infiltrated and the samples were analyzed for RLUC activity at 3 dpi. (C) The effect of other PVA proteins on HCPro^WD^’s capacity to suppress RNA silencing. *Agrobacterium* carrying YFP expression construct (OD_600_ = 0.01) and pHG-GFP (OD_600_ = 0.4) were co-infiltrated as in (B) with HCPro^WD^ / HCPro^WT^ and PVA^ΔHCPro^ (OD_600_ = 0.1). YFP fluorescence was quantitated from leaf discs collected at 4 dpi using a 96-well plate reader at Ex/Em 500/530 nm [51]. (D) Suppression of RNA silencing targeting PVA RNA-derived RLUC expression. The experimental setup was similar to that of (B). *Agrobacterium* carrying PVA^ΔHCPro^ was infiltrated at OD_600_ = 0.05. RLUC activities were measured from samples collected at 3dpi. The number of plants equals 4 (n = 4) in (B), n = 9 in (C) and n = 6 in (D). Different letters above the bars indicate a statistically significant difference (student’s t-test *P < 0.05). (E, F) Variation in the amount of PGs during transient expression of HCPro^WD^ (E) and HCPro^WT^ (F) infiltrated at OD_600_ = 0.1. P0^YFP^ was co-expressed at OD_600_ = 0.1 to visualize PGs. Samples collected at 3 dpi were examined with an epifluorescence microscope under an FITC filter. (G) Amount of PGs induced by HCPro^WD^ / HCPro^WT^ overexpression. HCPro^WD^ / HCPro^WT^ were agroinfiltrated at OD_600_ = 0.1 and 0.3 as indicated, while P0^YFP^ was agroinfiltrated at OD_600_ = 0.1. The number of PGs per mm^2^ was calculated from nine independent areas. Asterisks denote statistical significance between the sample sets (student’s t-test *P < 0.05, ***P < 0.001). (H) TwinStrep tag-affinity purifications were carried out from leaf samples collected at 3 dpi from plants infiltrated with either PVA^WD-Strep-RFP^ or PVA^WT-Strep-RFP^ (OD_600_ = 0.1). The purification resulted in the pulldown of HCPro along with its *in vivo* binding partners. Purified proteins were analyzed by western blotting using α-HCPro, α-CI, α-SAMS and α-VPg antibodies, respectively. All the blots were made from the same gel by cutting the PVDF membrane into strips after the transfer. In addition, two lanes of the same gel were silver-stained. The low molecular weight bands marked with asterisk demonstrates equal loading of the HCPro^WD-Strep-RFP^ and HCPro^WT-Strep-RFP^ samples.

Ability to suppress RNA silencing is undoubtedly one of the most studied properties of HCPro. To assess whether the HCPro^WD^ mutation affected the silencing suppression capacity of HCPro we used a dsRNA-triggered silencing assay based on the simultaneous transient expression of a transgene and a hairpin targeting it (Fig 3A). We tested the ability of HCPro to rescue the expression of the silenced transgene. The study was carried out in three different contexts. In the first case, RLUC was expressed along with a hairpin-RLUC (pHG-RLUC) construct and either HCPro^WD^ or HCPro^WT^ was expressed to rescue RLUC expression (Fig 3B). Secondly, YFP and a hairpin targeting it (pHG-GFP) were expressed in the presence of HCPro^WD^ / HCPro^WT^ and PVA^ΔHCPro^. PVA^ΔHCPro^ was included to see if other PVA proteins could contribute to the silencing suppression process (Fig 3C). Finally, pHG-RLUC was employed to target RLUC expressed from PVA^ΔHCPro^ RNA and either HCPro^WD^ or HCPro^WT^ was expressed to rescue viral gene expression (Fig 3D). In all cases, HCPro^WT^ successfully recovered the transgene and viral expression while HCPro^WD^ did not. These experiments clearly demonstrated that HCPro^WD^ is deficient in dsRNA-triggered RNA silencing suppression.

In our earlier work, we proposed a role for HCPro in formation of PVA induced granules (PGs), and we suggested PGs have a role in suppression of RNA silencing [10]. Since HCPro^WD^ was unable to suppress RNA silencing, we next compared its PG formation efficiency to that of HCPro^WT^. For this we co-infiltrated leaves with either HCPro^WD^ or HCPro^WT^ and P0^YFP^, which is a visual marker for PGs [10], all at OD_600_ = 0.1. The extent of PG induction by the HCPro variants was calculated at 3 dpi. The amount of PGs was reduced by almost five-fold in the presence of HCPro^WD^ as compared to HCPro^WT^ (Fig 3E–3G), which showed that the impaired ability of HCPro^WD^ to act as a suppressor of RNA silencing correlated with its capacity to induce PG formation. Increasing the OD of infiltration (to OD_600_ = 0.3) did not increase granule count either for HCPro^WD^ or HCPro^WT^. Rather, a reduction in the granule count was noticed in the case of HCPro^WT^, indicating that the potency of the PG-inducing property of HCPro does not necessarily correlate with the increased protein amount (Fig 3G). No granules were detected in the control images where P0^YFP^ was expressed alone with 35S-GUS (S3 Fig).

### HCPro^WD^ is defective in forming PVA infection-associated high molecular weight (HMW) complexes

Previously we reported the formation of highly stable HMW complexes during PVA infection [12]. These complexes need HCPro to form and they contain several infection-associated host and viral proteins. We also suggested that these complexes play a role in PVA translation [12]. In this study, we tested if the HCPro^WD^ mutation changed HCPro’s ability to form these HMW complexes. For this, we adopted a similar strategy reported in Ivanov et al. [12] with minor modifications. *N*. *benthamiana* plants were infected with PVA^WD-Strep-RFP^ or PVA^WT-Strep-RFP^ at OD_600_ = 0.1. Since PVA^WD^ gene expression in systemic leaves was low (Fig 2C), samples were collected from the local leaves at 3 dpi. The affinity purification of HCPro^WD/WT-Strep-RFP^ was performed following the method described in [30]. The purified samples were analysed by probing the protein gel blots with antibodies recognizing some of the previously identified HCPro^WT^ interactors including S–adenosyl-L–methionine synthase (SAMS), VPg and CI [12]. The HMW bands detected in the immunoblots had a similar electrophoretic mobility as those in the silver stained gel (Fig 3H). Based on visual inspection the amounts of SAMS, CI, and VPg were reduced in the HCPro^WD^ samples compared to HCPro^WT^ (Fig 3H). LC-MS/MS analysis of the same samples confirmed the western blot results (S2 Table). Interestingly, the silver stained gel showed an overall enrichment of complexes in the HMW region in HCPro^WT^ samples compared to HCPro^WD^. Equivalent loading of the samples is demonstrated by the equal intensity of a 55 kDa band in the silver stained gel (left panel Fig 3H).

### HCPro^WD^ is unable to sequester WD40 domain-containing host protein VCS into PGs

Earlier, Hafrén et al. [10] established VCS as a component of HCPro-induced PGs and an important factor in PVA infection. Furthermore, systemic infection, PVA translation, and coat protein (CP) accumulation were all affected by VCS knockdown [10]. Since VCS is a WD40 repeat-containing protein and the effects of low VCS levels on infection were parallel with the defects observed in PVA^WD^ infection, we decided to assess whether the HCPro^WD^ -binding site is needed to connect it to VCS. To this end, we tested whether VCS localized to PG structures in the presence of HCPro^WD^. For this experiment, we first generated VCS expression constructs. *N*. *benthamiana* annotated transcript data available on the Sol Genomics Network (http://solgenomics.net) document different variants of VCS. We used BLAST searches to identify three genes in the *N*. *benthamiana* genome with circa 50% identity to the *Arabidopsis thaliana* VARICOSE (Q9LTT8): VCS-A (Niben101Scf00654g02003.1), VCS-B (Niben101Scf39216g00007.1) and VCS-C (Niben101Scf21107g00015.1). We generated expression constructs of these three *N*. *benthamiana* genes along with their tagged versions (see Fig 1F).

**Fig 4:**
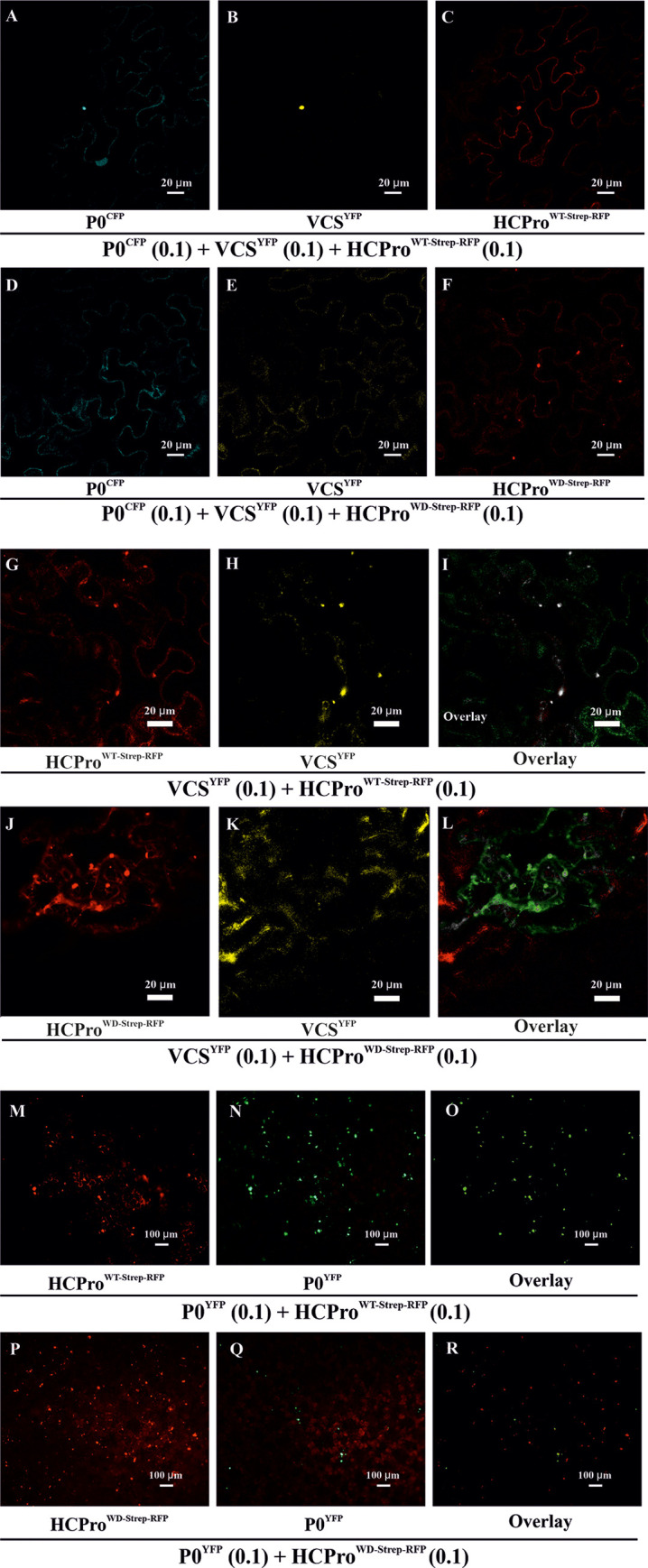
HCPro^WD^ is defective in recruiting VCS to PGs. (A-F) Localization of Strep-RFP-tagged -HCPro, YFP -tagged-VCS and CFP tagged -P0 in *N*. *benthamiana* epidermal cells. *Agrobacterium* carrying either HCPro^WT-Strep-RFP^ or HCPro^WD-Strep-RFP^ was infiltrated at OD_600_ = 0.1, while *Agrobacteria* harboring VCS-A^YFP^, VCS-B^YFP^ and VCS-C^YFP^ were each infiltrated at OD_600_ = 0.1 (together denoted as VCS^YFP^). *Agrobacterium* harboring P0^CFP^, infiltrated at OD_600_ = 0.1, was expressed as another marker for PGs. Samples were analyzed at 3 dpi by confocal laser scanning microscopy in sequential scanning mode. (A) P0^CFP^, (B) VCS^YFP^, (C) HCPro^WT-Strep-RFP^, (D) P0^CFP^, (E) VCS-^YFP^ and (F) HCPro^WD-Strep-RFP^. (G-L) Localization of HCPro^WT/WD-Strep-RFP^ and VCS^YFP^ and their overlays in *N*. *benthamiana* epidermal cells. Experimental details are otherwise the same as in Fig 4A–4F, except for the absence of P0^CFP^. (G) HCPro^WT-Strep-RFP^, (H) VCS-^YFP^ and (I) Overlay of HCPro^WT-Strep-RFP^ and VCS^YFP^. (J) HCPro^WD-Strep-RFP^, (K) VCS^YFP^ and (L) Overlay of HCPro^WD-Strep-RFP^ and VCS^YFP^. Degree of co-localization between HCPro^WT-Strep-RFP^ / HCPro^WD-Strep-RFP^ and VCS^YFP^ is calculated from representative images and presented in S6 Fig. (M-R) Epifluorescence microscopy showing the distribution of HCPro^WT/WD-Strep-RFP^ and P0^YFP^ within a representative area of examination. HCPro and P0^YFP^, which both typically co-localize in PGs, were co-infiltrated to visualize the PGs. The experiment was conducted similarly as Fig 3E and 3F. The only exception was that HCPro^WT/WD-Strep-RFP^ was used instead of HCPro^WT/WD^. Images were acquired at 3 dpi with a 10X objective. HCPro^WT/WD-Strep-RFP^ was visualized under an RFP filter (M, P respectively). P0^YFP^ from the corresponding sample sets was visualized under the FITC filter (N, Q respectively), and their overlays are presented in (O, R), respectively.

Next we infiltrated *N*. *benthamiana* leaves with either RFP-tagged HCPro or the mutated version (HCPro^WT-Strep-RFP^ / HCPro^WD-Strep-RFP^ at OD_600_ = 0.1) to induce PGs, YFP-tagged VCS (VCS-A^YFP^, VCS-B^YFP^ and VCS-C^YFP^ each at OD_600_ = 0.1 cumulatively denoted by VCS^YFP^), and another PG marker P0^CFP^ (OD_600_ = 0.1). Their localization was monitored via confocal microscopy at 3 dpi. The results reveal that HCPro^WT^ was able to sequester VCS into the PGs. Co-localization of the PG marker P0^CFP^ [10] in the same spot validated them as *bona fide* PGs. Unless the threshold effect prevented the detection of an overlap, HCPro^WD-Strep-RFP^ -induced cytoplasmic foci did not co-localize with either VCS^YFP^ or P0^CFP^ (Fig 4A–4F). Pairwise overlaps of the fluorescence signals corresponding to Fig 4A–4F are shown in S4 Fig, and the accumulations of HCPro^WD/WT-Strep-RFP^ and VCS^YFP^ related to the Fig 3A–3F is shown in the S5 Fig. Although a small difference could be detected in the accumulation levels of HCPro^WT^ and HCPro^WD^, they were still comparable.

### HCPro^WD^ mutation impairs HCPro-VCS co-localization

Next, we compared the extent of VCS co-localization with HCPro^WD^ and HCPro^WT^. For this we used similar experimental setup as Fig 4A–4F, except that P0^CFP^ was omitted. Confocal microscopy images from 3 dpi samples clearly show that HCPro^WT-Strep-RFP^ and VCS^YFP^ accumulated together to distinct foci with near perfect co-localization between these proteins (Fig 4G–4I). On the other hand, while HCPro^WD-Strep-RFP^ accumulated in distinct foci (Fig 4J), VCS^YFP^ remained diffuse in the cytoplasm (Fig 4K). The overlay image (Fig 4L) shows reduced co-localization between VCS^YFP^ and HCPro^WD-Strep-RFP^. The amount of co-localization with VCS^YFP^ was calculated from the confocal image data for both HCPro^WT-Strep-RFP^ and HCPro^WD-Strep-RFP^ (S6 Fig). Approximately a 48% reduction was observed with the HCPro^WD^ mutant. The relative expression levels corresponding to Fig 4G–4L of HCPro^WD-Strep-RFP^ and HCPro^WT-Strep-RFP^ and that of VSC^YFP^ in the presence of both HCPro versions were comparable (S7 Fig). These data along with Fig 4A–4F advocate the importance of the WD40 domain-binding motif in HCPro-VCS co-localization *in planta*.

### HCPro^WD^ mutation impairs HCPro’s ability to induce functional PGs

The low level of co-localization between VCS and HCPro^WD^ was an interesting finding especially since we still observed plenty of HCPro^WD-Strep-RFP^ in PG-like foci (Fig 4F–4J). Epifluorescence microscopy revealed a more than 75% coincidence of HCPro^WT^ with PG marker P0^YFP^ (S8A Fig, Fig 4M–4O). The amount of co-localization between HCPro^WD^ and P0^YFP^ was ten-fold lower (S8A Fig). This shows that only a marginal portion of HCPro^WD-Strep-RFP^ containing foci were PGs, which typically contain P0^YFP^as found in Hafrén et al.[10]. To show the lack of co-localization of HCPro^WD^ and P0^YFP^ in detail, an image of a single intact cell is provided in (S8B–S8D Fig). A reference PG induced by HCPro^WT^ is shown in (S8E–S8G Fig). Cumulatively the confocal and epifluorescence microscopy results indicate that the WD domain-interacting motif in HCPro is required for HCPro’s ability to recruit vital granule components to form functional PGs.

### HCPro-VCS association in HMW complexes is affected by the HCPro^WD^ mutation

**Fig 5:**
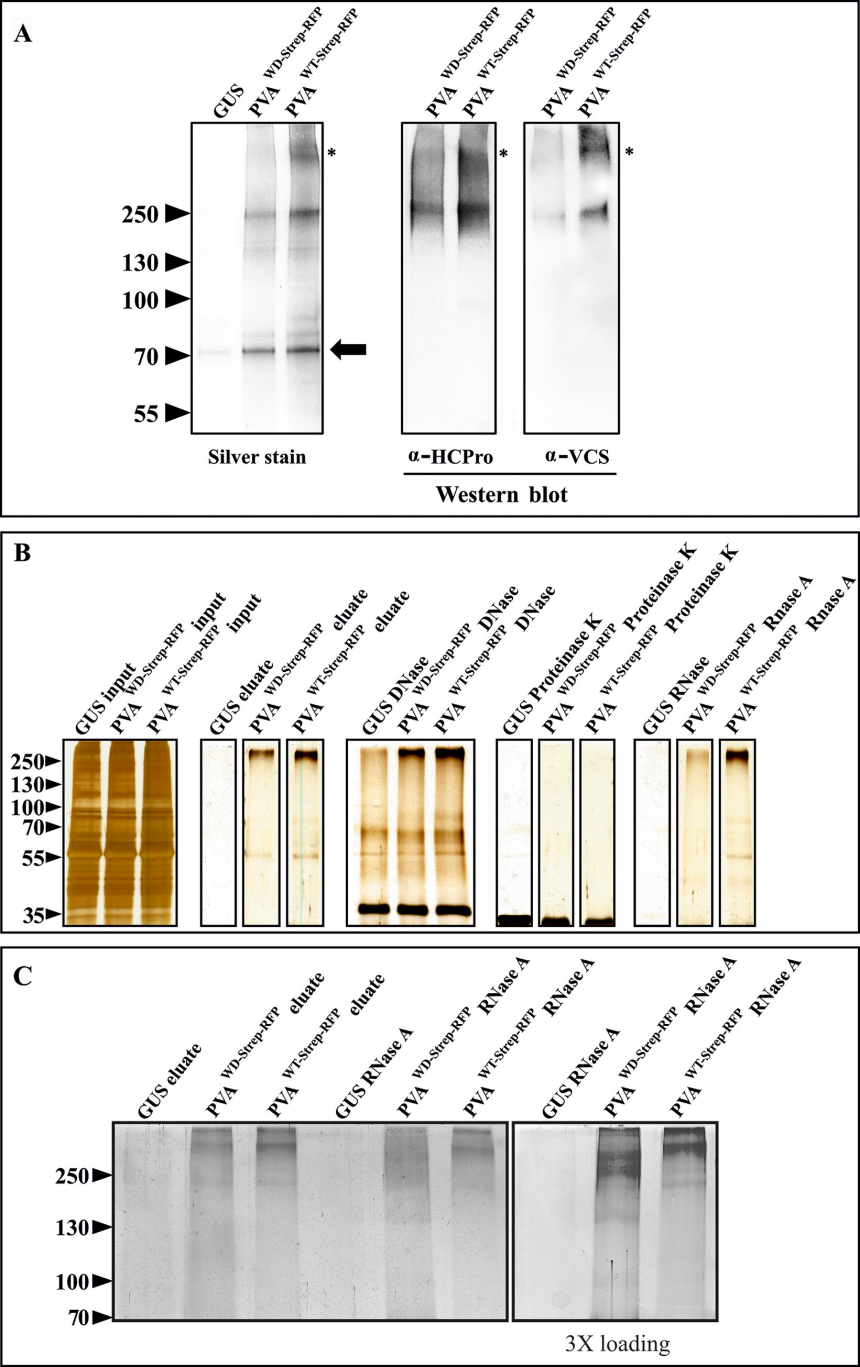
The WD mutation weakens VCS association with HCPro in HMW RNP complexes and the stability of the complexes. (A) HCPro and VCS assemble into HMW complexes during infection. For affinity purification, PVA^WD- Strep-RFP^ / PVA^WT-Strep-RFP^ constructs were agroinfiltrated at OD_600_ = 0.1 and the infected local leaves were sampled at 3 dpi. Both HCPro and VCS antibodies recognized HMW bands with a similar electrophoretic mobility in the purified HCPro^WT-Strep-RFP^ samples. The uppermost HCPro and VCS containing band was missing in HCPro^WD-Strep-RFP^ samples (marked with asterisks) both in the silver-stained gel and in the western blots. Equal loading is demonstrated in the silver-stained gel by the low molecular weight bands present in the samples (marked with an arrow). All the samples were run in the same gel. (B) Validation of the HCPro^WD-Strep-RFP^ / HCPro^WT-Strep-RFP^ affinity purification procedure by SDS PAGE and silver staining. HMW complexes are visible in the HCPro^WD/WT-Strep-RFP^ eluates. The purified products were treated with DNase, Proteinase K and RNase A and subsequently subjected to SDS-PAGE. The silver-stained gels demonstrate the stability of the purified HMW complexes. (C) RNase degradation of HCPro-associated HMW RNP complexes. To achieve maximum separation of the HMW RNP complexes, samples similar to those in A) were loaded in varying quantities and run for a longer time in a 10% SDS-PAGE gel. In all the cases, PVA^WD-Strep-RFP^ and PVA^WT-Strep-RFP^ samples were loaded equally similarly as in A). Samples presented in the right panel are similar to those shown in the adjacent left panel except that three folds more was loaded to visualize the effect of RNase A treatment on complexes in PVA^WD-Strep-RFP^ samples. All the samples in C) were part of the same gel but the right panel was developed for a longer time than the left panel during silver staining.

In order to demonstrate the association of HCPro and VCS in HMW complexes formed during PVA infection in *N*. *benthamiana*, we affinity purified Strep-tagged HCPro and tested the samples for the co-immunoprecipitation of VCS. In the affinity purification experiment, the plants were infected with PVA^WD-Strep-RFP^ or PVA^WT-Strep-RFP^ at OD_600_ = 0.1. Samples were collected from local leaves at 3 dpi and the affinity purification of HCPro^WD/WT-Strep-RFP^ was performed following the method described in [30]. The presence of HCPro and VCS in the purified samples was investigated by immunoblotting with α-HCPro and α-VCS antibodies (Fig 5A). Equivalent loading of the purified HCPro^WD-Strep-RFP^ and HCPro^WT-Strep-RFP^ samples is demonstrated by the equal intensities of low molecular weight bands, the major one marked with an arrow at the ~70 kDa region in the silver stained gel. The lack of any prominent bands in the GUS control indicated a negligible amount of non-specific interactions with Strep-Tactin matrix during purification. Both HCPro and VCS bands were detected in the HMW- region (>250 kDa). In contrast to the HCPro^WT-Strep-RFP^, top-most band containing VCS (marked with an asterisk) was missing in HCPro^WD-Strep-RFP^ samples. Interestingly, the top-most band was also absent in the silver stained gel and drastically reduced in the α-HCPro immunoblot in the HCPro^WD-Strep-RFP^ pull-down samples. A comparison between the input and the eluted samples indicated specific enrichment of HMW complexes upon purification (S9 Fig). Additionally, it further validated the specificity of disappearance of the top-most VCS band from HCPro^WD-Strep-RFP^ purified samples. Nevertheless, HMW complexes approximately 250 kDa in size were still present in HCPro^WD-Strep-RFP^ samples. This suggests that the mutation in the WD40 domain-interacting motif of HCPro affected but did not completely abolish the formation of HCPro- and VCS-containing HMW-complexes during PVA infection.

### HMW complexes formed during PVA^WD Strep-RFP^ infection are sensitive to RNase treatment

Results presented in Fig 3H and Fig 5A indicated compositional differences between HCPro^WD^ and HCPro^WT^ -containing HMW complexes. Several HMW components such as CI, VPg, SAMS and VCS were specifically reduced in HCPro^WD^ samples. Next, we probed into the stability of the HMW complexes. For this, we treated the affinity-purified samples with DNase, proteinase K or RNase A followed by SDS-PAGE analysis. Both HCPro^WD^ and HCPro^WT^–containing HMW complexes resisted DNase treatment whereas proteinase K degraded them completely (Fig 5B). Intriguingly, RNase A affected HCPro^WD^-HMW complexes, while it did not affect the HCPro^WT^ ones (Fig 5B). A bulk reduction of the HCPro^WD^-HMW complexes upon RNase A treatment suggested that they are ribonucleoprotein (RNP) complexes. To probe into this phenomenon further, we ran the RNase A degraded complexes longer in the gel and optimized the loading quantity to get a better look on the degraded complexes (Fig 5C). Smearing of the bands rather than the presence of individual low-molecular weight proteins suggests the existence of loosely bound multiprotein complexes migrating at the HMW region even when the RNA fraction exposed to RNase treatment had been degraded (Fig 5C). The data suggest that the impaired assembly of HCPro^WD^ and the other HMW components compromised the stability of these complexes rendering the RNA prone to degradation by RNase A.

### VCS knockdown reduces PVA^WT^ gene expression and accumulation to the level of PVA^WD^

**Fig 6:**
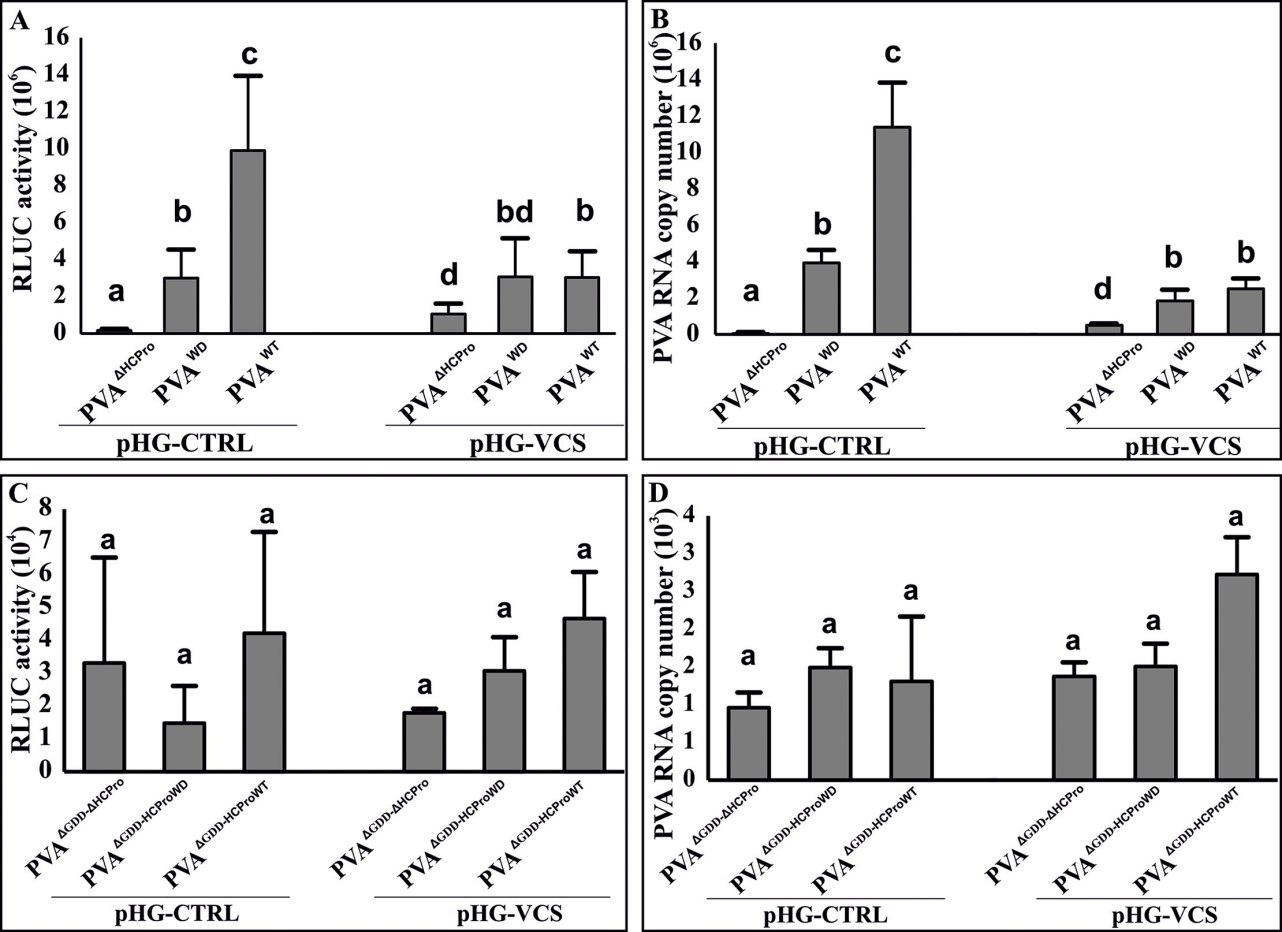
VCS knockdown brings down PVA^WT^ expression level to that of PVA^WD^. (A, B) *N*. *benthamiana* leaves were infiltrated with *Agrobacterium* carrying a silencing vector (pHG-VCS) expressing a hairpin RNA targeting VCS (OD_600_ = 0.4). An empty Hellsgate plasmid (pHG-CTRL) was used as a control. *Agrobacterium* carrying PVA^ΔHCPro^, PVA^WD^ and PVA^WT^ constructs were co-infiltrated (OD_600_ = 0.01) with *Agrobacterium* carrying 35S-FLUC (OD_600_ = 0.01) for normalization of the virus-derived RLUC values. Normalized RLUC (A) and PVA RNA (B) levels were quantitated at 4 dpi. (C) Normalized RLUC and (D) RNA values derived from non-replicating PVA RNAs: PVA^ΔGDD-ΔHCPro^, PVA ^ΔGDD-HCProWD^ and PVA ^ΔGDD-HCProWT^. VCS silencing was done similarly as in (A). *Agrobacterium* carrying PVA^ΔGDD-ΔHCPro^, PVA ^ΔGDD-HCProWD^ and PVA ^ΔGDD-HCProWT^ constructs were co-infiltrated (OD_600_ = 0.05) with *Agrobacterium* carrying 35S-FLUC (OD_600_ = 0.01) for normalization of the virus-derived RLUC values. Samples were collected at 3 dpi. The number of plants per experiment was 6 in (A, B) and 3 in (C). Statistical significance was assessed using student’s *t*-test. Different letters above the bars indicate a statistically significant difference (*P* < 0.05).

We considered the WD-mutation -mediated defect in HCPro-VCS association could be a possible reason behind the aberrant behaviour of HCPro^WD^ and sub-optimal performance of PVA^WD^. To test this idea we characterized the role of HCPro-VCS interaction in PVA infection. We studied how limited VCS availability affected PVA^ΔHCPro^, PVA^WD^ and PVA ^WT^ infection. For this, we transiently knocked down VCS by infiltrating leaves with a hairpin construct targeting VCS (pHG-VCS). PVA^ΔHCPro^, PVA^WD^ and PVA ^WT^ expression levels were then measured both in the control and VCS-silenced background. VCS silencing was validated via reverse transcription-polymerase chain reaction (RT-PCR) (S10 Fig). Following a pattern similar to Fig 2A, PVA^WD^ gene expression level was approximately 5-fold lower than that of PVA^WT^ (Fig 6A) and approximately 20-fold higher than that of PVA^ΔHCPro^ in the control plants. On the other hand, in VCS knock-down experiments PVA^WT^ gene expression decreased to the level of PVA^WD^, while that of PVA^WD^ remained unaffected. Interestingly, gene expression of PVA^ΔHCPro^ increased upon VCS downregulation (Fig 6A), showing that in the absence of HCPro, VCS is an antiviral protein. vRNA levels followed the same pattern as RLUC expression throughout this experiment (Fig 6B). In contrast to the replicating viruses, RLUC and vRNA were expressed at similar levels from the replication-deficient variants of PVA (PVA^ΔGDD-ΔHCPro^, PVA^ΔGDD-HCProWD^ and PVA^ΔGDD- HCProWT^) and did not change upon VCS silencing (Fig 6C and 6D). Thus, the VCS-HCPro interaction is especially important for the functions of the replicating PVA RNA. Interestingly, VCS seemed to promote PVA^WT^ infection in the presence and restrict it in the absence of HCPro. PVA^WD^ was insensitive to VCS knockdown while PVA^WT^ decreased to the level of PVA^WD^ supporting the role of impaired HCPro-VCS interaction in the reduced PVA^WD^ infection. Our interpretation of this result is that HCPro has the capacity to convert VCS from an antiviral protein to the support of the PVA infection.

### VPg, HCPro and VCS together promote PVA translation

We proposed in our previous paper [12] that the presence of HCPro and AGO1 on polysomes may indicate that RNA silencing-related translational repression is involved in PVA infection and HCPro is required to relieve it. Also, we reported a role for VPg and HCPro in PVA translational regulation, and proposed the possible involvement of VCS in VPg-mediated translational enhancement [10, 31, 32]. In this study, we probed into the role of VCS in PVA translation by overexpressing either VPg, VCS or both together with PVA^ΔGDD-ΔHCPro^, PVA^ΔGDD-HCProWD^ and PVA^ΔGDD-HCProWT^. The replication-deficient PVAs were used in this experiment to narrow down the effects of VCS, VPg and HCPro to aspects pertaining to PVA translation. More vigorous protein production from an unaltered RNA concentration, which affects the ratio of the protein and its mRNA, has been regarded as an indicator of release from translational repression [33]. Therefore, we calculated the RLUC/vRNA ratios from PVA^ΔGDD-ΔHCPro^, PVA^ΔGDD-HCProWD^ and PVA^ΔGDD-HCProWT^ upon VPg and VCS overexpression. In this experiment, all the constructs were co-infiltrated and samples were collected at 3 dpi. The absolute values for RLUC activities and RNA levels were quantitated and are presented in S11A and S11B Fig. Overexpression of VCS was validated via qPCR (S11C and S11D Fig).

**Fig 7:**
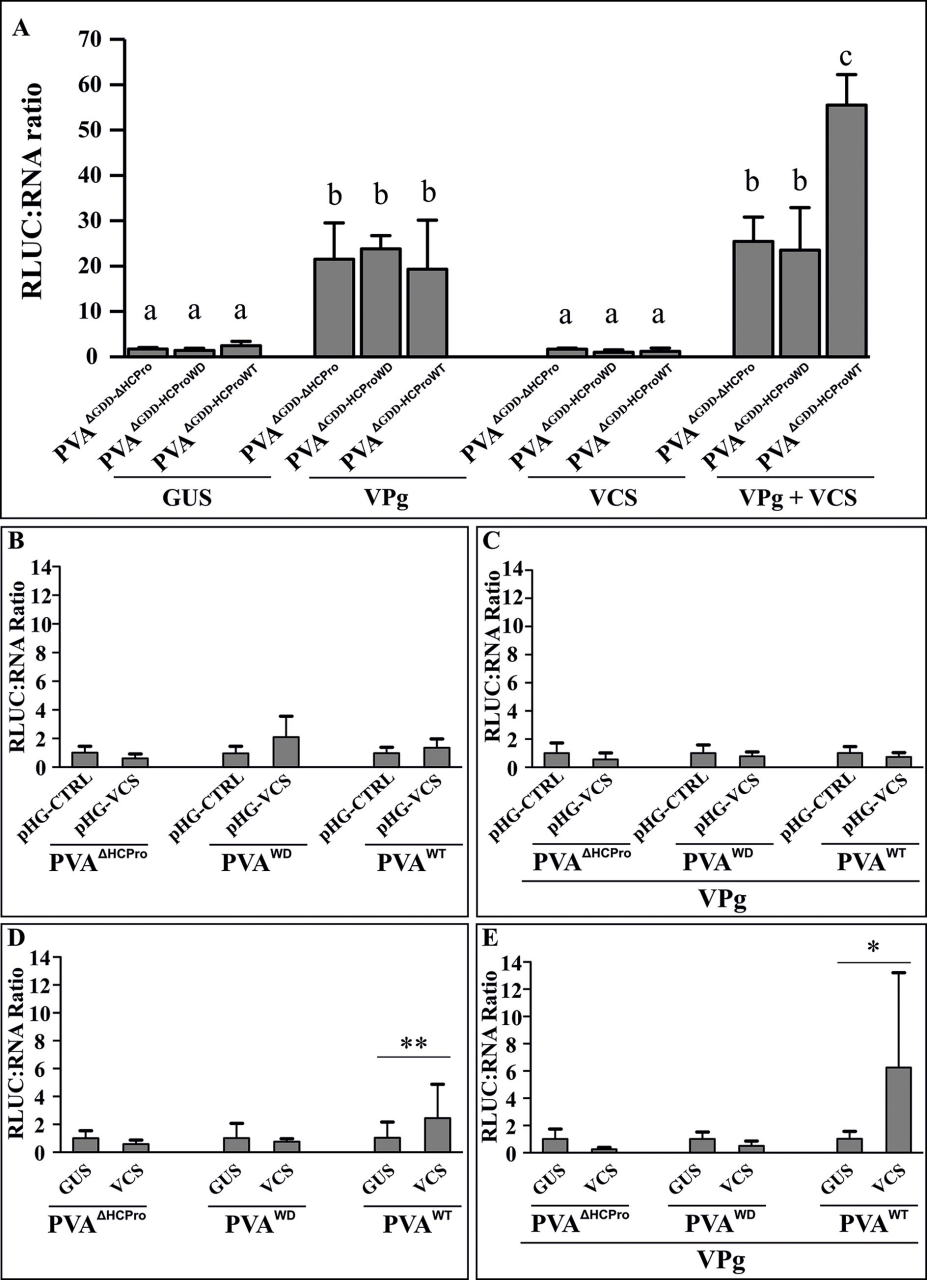
Both VPg and VCS-HCPro interaction promote PVA translation. (A) RLUC/vRNA ratio of non-replicating variants of PVA RNA (PVA^ΔGDD-ΔHCPro^, PVA^ΔGDD-HCProWD^ and PVA^ΔGDD-HCProWT^) was quantitated during VPg, VCS and VPg + VCS overexpression. All virus constructs were infiltrated at OD_600_ = 0.05 and all overexpression constructs at OD_600_ = 0.25. VCS overexpression was initiated with a mix of *Agrobacterium* carrying VCS-A, VCS-B and VCS-C constructs each at OD_600_ = 0.25. Samples for the dual luciferase assay and RNA quantification via qPCR were collected at 3 dpi. Statistical significance was assessed using student’s *t*-test (*P < 0.05). Different letters above the bars indicate a statistically significant difference. (B—D) Fold change in the RLUC/vRNA ratio in replicating variants of PVA- (PVA^ΔHCPro^, PVA^WD^ and PVA^WT^) upon VCS-silencing (B, C) and overexpression (D, E). The ratios were determined both without (B, D) and with (C, E) overexpressed VPg. All PVA constructs were infiltrated at OD_600_ = 0.01. The silencing constructs were infiltrated one day before virus infiltration at OD_600_ = 0.4, whereas the overexpression constructs were co-infiltrated with PVA constructs. VPg was infiltrated at OD_600_ = 0.4. The VCS overexpression set contained a mix of VCS-A, VCS-B and VCS-C each infiltrated at OD_600_ = 0.1. All samples were collected at 4 dpi. The concentration of infiltrated *Agrobacterium* between different samples in (A-E) was equalized with *Agrobacterium* carrying either pHG-CTRL or GUS. The number of plants per experiment was 4 in (A), 6 in (B, C) and 5 in (D, E). Statistical significance between the sets is denoted by asterisk (student’s t-test *P < 0.05, **P < 0.01).

Irrespective of which HCPro variant was present, VPg enhanced the RLUC/vRNA ratio similarly for all of the replication-deficient PVAs (Fig 7A). The absolute RLUC activity measurements show that both PVA^ΔGDD-HCProWD^ and PVA^ΔGDD-HCProWT^ are equally enhanced by VPg (S11A Fig). This suggests that VPg-mediated enhancement of translation does not require an intactWD40 domain-binding motif in HCPro. We further validated this by supplementing non-replicating PVA^ΔGDD-ΔHCPro^ with VPg together with either GUS (OD_600_ = 1), HCPro^SDM^ (OD_600_ = 1), HCPro^4EBM^ (OD_600_ = 1), HCPro^WD^ (OD_600_ = 0.3), or HCPro^WT^ (OD_600_ = 0.3). VPg did not enhance translation in the presence of HCPro^SDM^ and HCPro^4EBM^. However, in the presence of both HCPro^WD^ and HCPro^WT^, VPg caused a similar extent of RLUC enhancement (S12 Fig), which further confirmed that the HCPro^WD^ mutation does not prevent the effect of VPg on PVA translation, whereas HCPro^SDM^ and HCPro^4EBM^ mutants do. Overexpression of VCS alone did not have a significant effect on the RLUC / vRNA ratio of any replication-deficient PVA. Finally, co-expression of VPg and VCS increased the RLUC/vRNA ratio of PVA^ΔGDD-HCProWT^ significantly more than that of PVA^ΔGDD-ΔHCPro^ and PVA^ΔGDD-HCProWD^. Both PVA^ΔGDD-ΔHCPro^ and PVA^ΔGDD-HCProWD^ performed similarly in the simultaneous overexpression of VPg and VCS. In fact, here the addition of VCS did not change the situation where VPg alone was overexpressed. Taken together, HCPro and VCS need to interact in order to achieve full translational benefit generated by the combined effect of VPg and VCS (Fig 7A).

Next we studied the effect of VPg and VCS on the translation of replicating PVAs. The RLUC/vRNA ratios of PVA^ΔHCPro^, PVA^WD^ and PVA^WT^ were calculated from VCS knock-down and overexpression experiments. In addition, we included transiently overexpressed VPg in the experiment to study its contribution to the RLUC/vRNA ratio with and without VCS. Samples were collected from the infiltrated leaves at 4 dpi. Silencing and overexpression of VCS were validated via qPCR (S13 Fig). After measuring RLUC activity and vRNA levels (S14 Fig) we calculated the RLUC/vRNA ratio for each sample. VCS knock-down either in the presence or in the absence of VPg did not significantly alter the RLUC/vRNA ratio of any PVA variant (Fig 7B and 7C). A small increase in the RLUC/vRNA ratio was detected upon VCS overexpression in plants infected with PVA^WT^ (Fig 7D). This ratio increased further when both VCS and VPg were co-expressed in PVA^WT^ infected plants (Fig 7E). Similar to the replication-deficient PVAs (S11A and S11B Fig), the change in the ratios caused by simultaneous overexpression of VPg and VCS were due to reduced vRNA accumulation and unaffected RLUC. While active PVA translation benefits from the collaboration of VPg, VCS and HCPro, the reduced RNA amount during VCS overexpression may indicate that other viral processes become unbalanced.

### VCS-HCPro interaction is required for particle formation

**Fig 8:**
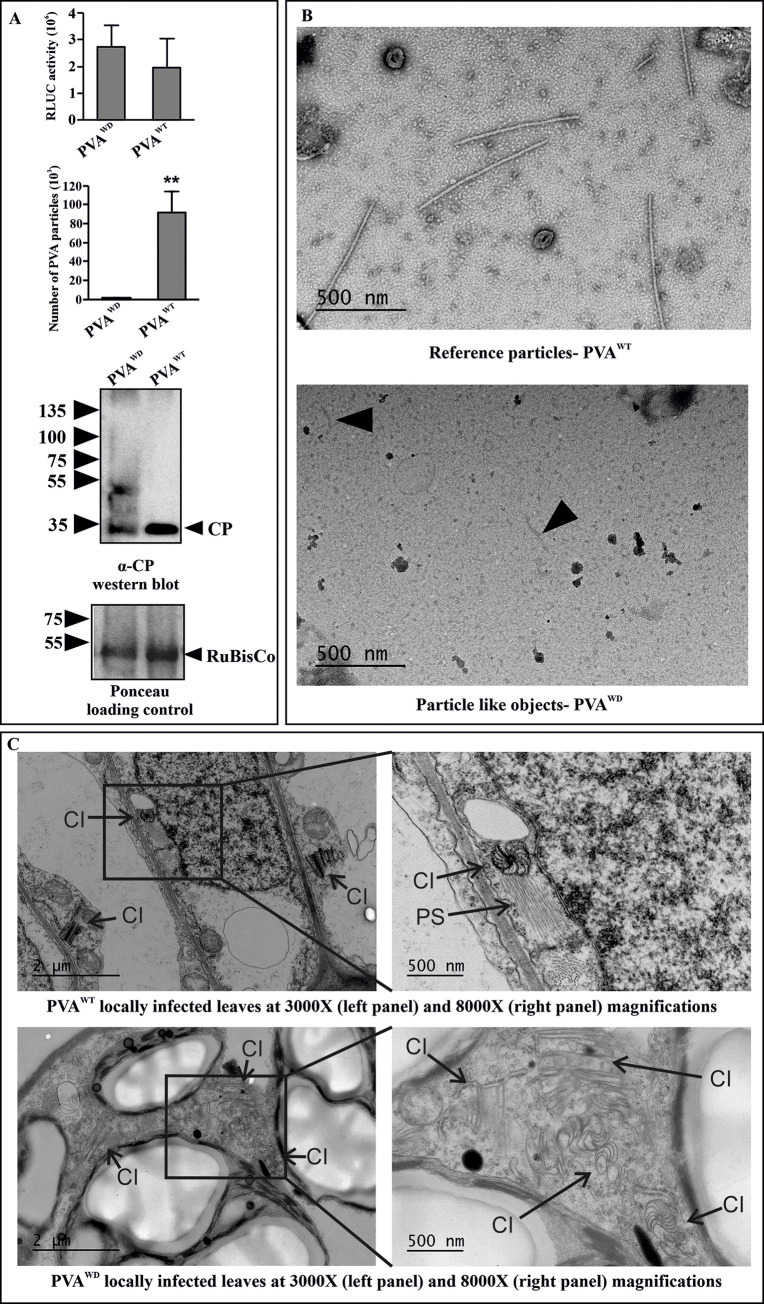
PVA^WD^ is defective in particle formation. (A) PVA particle accumulation in PVA^WD^ and PVA^WT^ infected local leaves at 3 dpi, as measured by immunocapture qRT-PCR. *N*. *benthamiana* plants were agroinfiltrated with PVA^WD^ (OD_600_ = 1) and PVA^WT^ (OD_600_ = 0.1) to ensure comparable RLUC expression level between PVA^WD^ and PVA^WT^ (upper panel). The corresponding particle numbers and CP accumulation visualized by western blotting with anti-CP antibodies are presented in the middle and lower panels, respectively. (B) Electron microscopy images from the same samples as in A) showing normal virus particles formed by PVA^WT^. No particles were found in PVA^WD^, only few deformed particle-like structures were observed (marked by an arrow in the lower electron micrograph). The number of plants per experiment was 3. Statistical significance was assessed using student’s *t*-test (*P < 0.05). (C) Both PVA^WT^ and PVA^WD^ infections were allowed to spread in the local leaves until 9 dpi, and EM images of the infected tissues were taken.Pinwheel inclusions (marked as ‘CI’) were observed. However, the pinwheels from PVA^WT^ were associated with stacks of particles (marked as ‘PS’) in vicinity while those from PVA^WD^ were devoid of them.

The importance of VCS for systemic infection and CP accumulation was proposed in Hafren et al. [10]. Here we show that in PVA^WD^ viral gene expression remained low in systemic leaves suggesting that very little of vRNA reached the upper leaves (see Fig 2C). Since systemic movement is often associated with the formation of stable virions [34–38], we next tested the ability of PVA^WD^ to form particles or virus-like particles (VLPs). For this, we sampled locally infected leaves at 3 dpi. In order to obtain equivalent infection PVA^WD^ was infiltrated in a higher amount (OD_600_ = 1) compared to PVA^WT^ (OD_600_ = 0.1). Immunocapture (IC)-qRT-PCR was performed to measure the abundance of vRNA assembled into VLPs or virions. Although RLUC activities were broadly similar, a drastic reduction in the amount of particles was noticed in PVA^WD^-infected plants as compared to PVA^WT^-infected plants (Fig 8A). CP accumulation was validated via western blotting. A slight reduction in the intensity of the CP band was observed in PVA^WD^ (Fig 8A). To visually inspect the particles, we extracted the particles in phosphate buffer and subjected them to transmission electron microscopy. Virus particles were abundantly detected in PVA^WT^-infected samples but none were observed in PVA^WD^-infected samples. A few short and loose particle-like structures were occasionally detected; however, they were virtually indistinguishable from imaging artefacts (Fig 8B). Taken together, IC-qRT-PCR results coupled with the electron microscopy study provided solid evidence that either PVA^WD^ does not form particles or the particles formed are fundamentally defective.

**Fig 9:**
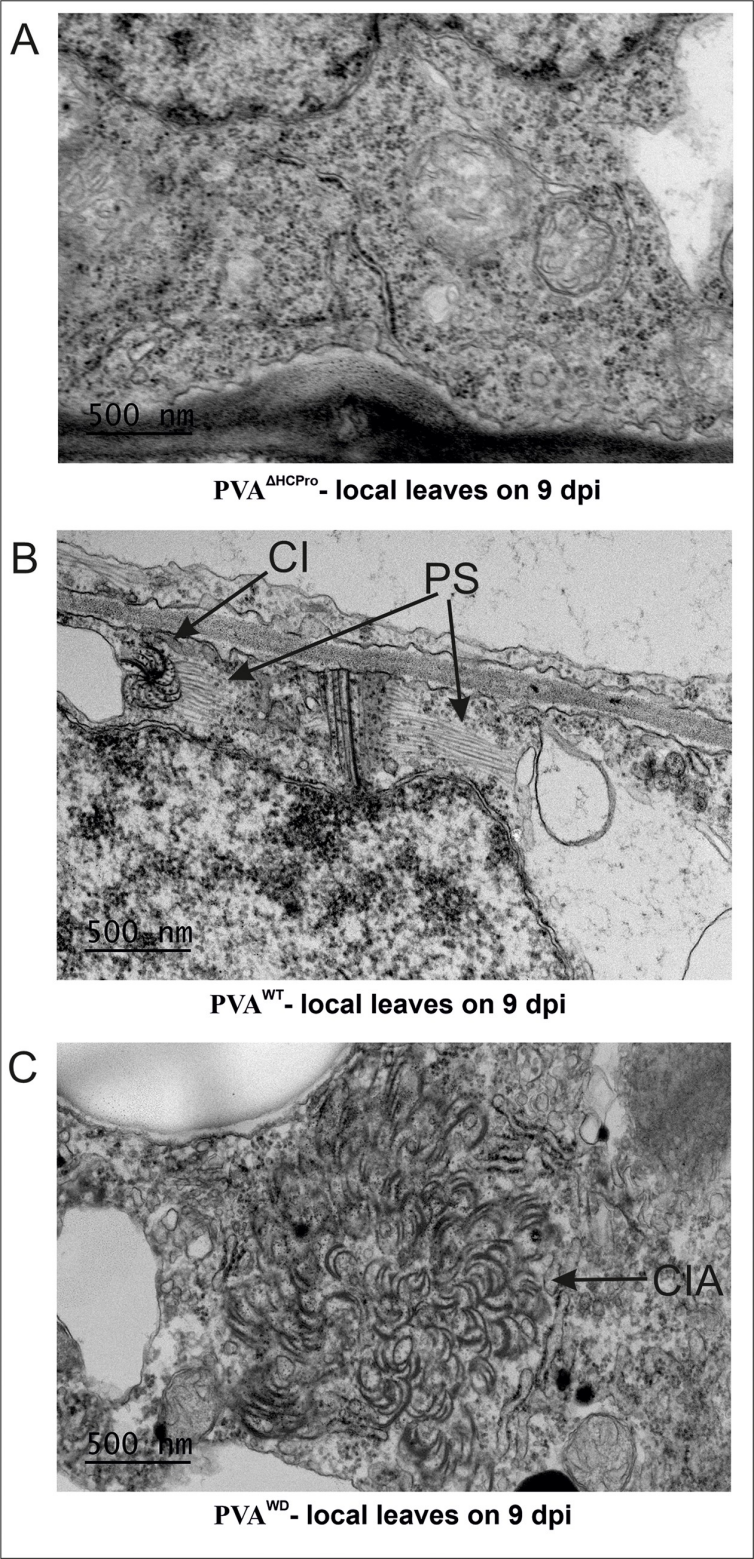
Homeostasis of pinwheel inclusions is disrupted in PVA^WD^ infection. (A) PVA^ΔHCPro^ infected leaf tissues did not show any pinwheel inclusions or any other visible symptoms of infection at 9 dpi. (B) PVA^WT^ infected cell showing characteristic pinwheel inclusions (CI) and associated particle stacks (marked as PS). (C) PVA^WD^ infected cells showing in several instances dense patches of pinwheel inclusions (marked as CIA). All the images were taken at 8000X magnification.

Finally, in order to probe into the nature of infection *in planta* we allowed the infection to spread in local leaves for 9 days, and visualized the leaves under transmission electron microscopy. Pinwheel structures characteristic for potyvirus infection were detected in both PVA^WT^ and PVA^WD^ samples (Fig 8C right panel). PVA^WD^ infected cells were, however, devoid of particle stacks observed in abundance in PVA^WT^ infected samples (Fig 8C left panel) [39]. While PVA^ΔHCPro^ failed to form pinwheel structures (Fig 9A) typically seen in PVA^WT^ infected cells (Fig 9B), PVA^WD^ infected cells showed dense aggregations of cylindrical inclusion protein (CI)-induced pinwheel structures (Fig 9C). Although it is not clear what the abundance of these pinwheel structures means, it could indicate that the HCPro^WD^ mutation disrupts the homeostasis of CI and pinwheel structures. It also shows that although the expression level of PVA^WD^ was high the infection was not able to launch robust particle formation.

## Discussion

**Fig 10:**
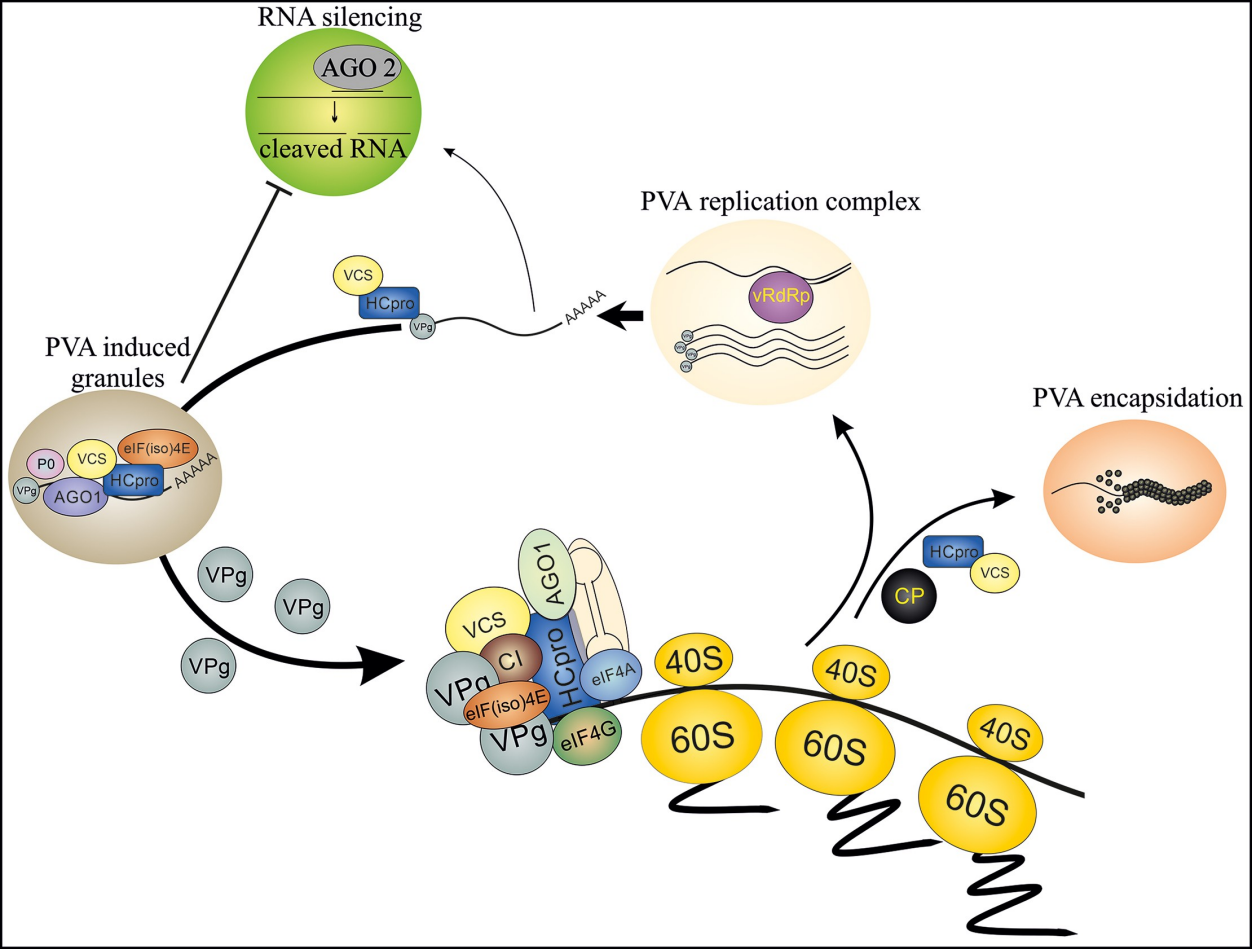
A model for HCPro-VCS interaction in PVA infection. We present here a hypothetical model of a core complex, which contains both HCPro and VCS and guides PVA RNA through the different stages of infection. We propose that after PVA RNA is released from the viral replication complex it connects with HCPro. HCPro then recruits VCS to assist in the formation of multiprotein complexes. The composition of these complexes probably varies depending on the infection stage. Interaction between HCPro and VCS is required for the formation of PGs, which form to suppress RNA silencing and to enable active PVA translation. Finally, the multiprotein complex formed around HCPro-VCS seals the PVA particles. This is essential for both the stability of PVA RNA within particles and the systemic spread of PVA infection.

The WD40-domain protein VCS has an important role in PVA infection and it associates with PGs [10]. In this study, we explored the interplay between HCPro and VCS *in planta* and its importance for achieving efficient infection. Using an HCPro mutant with a compromised capacity for sequestering VCS in infection-associated multiprotein complexes, and its lack of sensitivity to the effects of VCS in PVA infection, we show that this interaction is pivotal for PG formation, silencing suppression, efficient translation, PVA encapsidation and systemic infection. We propose a model of the PVA infection cycle highlighting the role of the HCPro-VCS interaction at various stages of the infection (Fig 10). In this model, we outline that during a successful infection several multiprotein complexes are formed around replicated PVA RNA. These complexes contain HCPro, VCS and both free and 5’ genome-linked VPg, along with other recruited proteins. We further propose that these complexes protect the viral RNA from degradation, support it in its essential functions and follow it all the way to the formation of stable particles.

WD40-domain proteins are known for their regulatory roles. They are involved for example in cell division, cell fate determination, gene transcription and mRNA modification [22]. According to the sequence analysis carried out using the WDSP web server motif predictor [20] *N*. *benthamiana* VCS-A and VCS-C are WD40-domain proteins that comprise five WD40 repeats, while VCS-B has six (http://www.wdspdb.com/wdsp/). In processing bodies VCS acts as an enhancer of mRNA decapping and associates with RNP complexes containing the decapping proteins DCP-1 and DCP-2 [27,40]. In addition, VCS has a role in miRNA-regulated translational repression [33]. Similar to its human analogue Ge-1/Hedsl, VCS is predicted to act as a scaffold for protein-protein interactions [27,41]. Furthermore, VCS and Ge-1/Hedsl have both been assigned pro-viral roles. Ge-1 is an important host factor in hepatitis C virus infection and its depletion significantly reduced viral protein and RNA accumulation [42]. Along the same lines, the down regulation of plant VCS reduced PVA infectivity [10].

The ‘AELPR’ motif of PVA HCPro identified in this study is shared by three WD domain -binding motifs conforming to the pattern [EDSTY].{0,4}[VIPLA][TSDEKR][ILVA]. Although there were other stretches of amino acids fulfilling this pattern within the PVA HCPro sequence, this motif was targeted because it is conserved within 119 potyviral HCPro sequences. In addition the motif localized to a surface-accessible disordered region (Fig 1B and 1C and S1 Fig), predicting that this region could participate in protein-protein interactions. Although the mutation site is near the protease domain, HCPro^WD^’s protease function was unaffected (Fig 2A). Therefore, we believe that a mutation within this loop structure did not disturb the overall HCPro structure. While the results of this study enable us to propose that this motif plays a role in the association between HCPro and VCS *in planta*, one or more of the other predicted WD-binding sites (see S1A Fig) may still permit weak interactions with VCS. This would be a possible explanation for the remaining degree of co-localization and co-purification of VCS with Strep-tagged HCPro^WD^. Moreover, other proteins present in the HCPro-containing HMW RNP complexes may also carry HCPro and VCS binding sites. In that case, the complexes would still retain HCPro and VCS, but in a loosely bound form.

The nature of PGs, including protein composition and comparison to processing bodies and stress granules are discussed in Hafrén et al.[10]. HCPro^WD^ overexpression produced abundant PG-like cytoplasmic foci. This feature was unique to this mutant as neither HCPro^WT^ (in this study and [10]) nor HCPro^SDM/4EBM^ [10] produced such aggregates. These aggregates may represent non-functional forms of PGs as most of them lacked vital PG components like VCS and P0, which are characteristic of HCPro^WT^-induced PGs as shown in [10], (also in S4 Fig and S8 Fig). This indicates that HCPro^WD^, though still able to form the foci, fails to sequester essential PG components leading to the accumulation of the non-functional cytoplasmic aggregates. A similar kind of malfunction was noted for the infection-associated HMW RNP complexes, to which HCPro^WD^ failed to recruit components like VPg and CI (Fig 3H and Fig 5A). Furthermore, the increased sensitivity of complex-bound RNA to RNase treatment (Fig 5B and 5C) indicated misassembled complexes with possibly loosely bound components. Our hypothesis is that during PVA^WT^ infection all components involved in the HCPro-induced multiprotein complexes co-operate to shelter vRNA from degradation. HCPro may provide the molecular cues while VCS likely forms a physical scaffold for the complex. Pinwheel structures are primarily attributed to CI but it is clear that assistance from other viral and host proteins is required to form them (reviewed in [43]). HCPro^WD^ was unable to recruit CI into HMW RNP complexes (Fig 3H and S2 Table). Although in PVA^WD^ infected samples CI formed dense patches of pinwheel aggregates uncommon to PVA^WT^ infection (Fig 9). Although we are unable to conclude if impaired HCPro-VCS interaction underlies this phenomenon, it provides another example of uncharacteristic multiprotein structures in a PVA^WD^ infection.

In our earlier studies we hypothesized that PGs are linked to the silencing suppression property of HCPro and, in order to achieve efficient infection, vRNA moves from replication to translation via PGs [10,44]. Active translation does not occur in visible PGs. The amount of PGs is actually reduced when PVA RNA is actively translated on polysomes [10]. Potyviral protein VPg is responsible for this regulation. Our recent data suggest that binding of PVA VPg to eIF(iso) 4E is a key factor in directing PVA RNA from PGs to active translation on polysomes [45]. VPg also protects PGs from degradation via autophagy [46]. This study further supports the hypothesis that PGs have a role in viral gene expression and in the protection of PVA RNA. First, HCPro^WD^ forms approximately five-fold less PGs compared to HCPro^WT^. On similar lines PVA^WD^ gene expression is approximately five-fold lower than PVA^WT^ gene expression. Second, HCPro^WD^ is deficient in hairpin-mediated RNA silencing suppression (Fig 3A–3D) and in recruiting VCS to PGs (Fig 4) VCS knockdown significantly reduces the amount of PGs induced by HCPro^WT^ [10], while in this study we demonstrated that VCS silencing brings PVA^WT^ expression down to the level of PVA^WD^ (Fig 6A and 6B). Cumulatively these results strengthen the argument that HCPro coordinates the recruitment of VCS in PGs through its WD40 domain-interaction motif.

This study successfully demarcates the role of HCPro in PVA translation from its role in silencing suppression. Earlier we demonstrated that VPg mediates the enhancement of RLUC expression from PVA RNA [31]. Furthermore, VPg-mediated translational regulation requires VCS and HCPro [10] and their interaction to optimally augment protein production from PVA RNA (Fig 7). PVA HCPro^SDM^ [10] is equivalent to the AS9 mutant well-characterized in TEV and TuMV [5,6,47], and PVA HCPro^4EBM^ is an eIF4E binding-deficient mutant of HCPro [13]. PVA HCPro^SDM^ and PVA HCPro^4EBM^ are both deficient in RNA silencing suppression [10]. Unlike in HCPro^SDM^ and HCPro^4EBM^, complementation was not fully lost in HCPro^WD^ (Fig 2B). Reduction in the complementation efficiency compared to the wild type HCPro is logical since HCPro^WD^ is deficient in silencing suppression. An interesting novel finding of this study is the demonstration that VPg-mediated translational enhancement could be complemented by HCPro^WD^ but not by HCPro^SDM^ and HCPro^4EBM^ (S12 Fig), which proposes that the role of VPg in translational enhancement does not rely on the HCPro-VCS interaction. Supporting this argument both HCPro^WD-Strep-RFP^ and HCPro^WT-Strep-RFP^ associated in monomeric form with polysomes purified from PVA^WD-Strep-RFP^ / PVA^WT-Strep-RFP^ infected *N*. *benthamiana* plants (S15 Fig and S1 Reference). A thorough understanding of translational regulation by these proteins is a subject of future studies.

Initially RISC-mediated translational repression was thought to be a unique feature of animal cells but lately it has been shown to be an antiviral mechanism also *in planta*. Iwakawa and Tomari [48] achieved strong repression of translation using sRNA with perfect complementarity to TEV 5’ UTR followed by an RLUC ORF. Furthermore, *Arabidopsis* VCS mutants show increased protein accumulation without a corresponding increase in mRNA level suggesting that VCS participates in translational repression [33]. The relevance of HCPro-VPg-VCS interactions in translational regulation was further supported by a significant upregulation in the RLUC / RNA ratio upon overexpression of VPg and VCS in PVA infection. Interestingly, in the PVA infection context, VCS overexpression decreased vRNA accumulation by approximately three-fold (S14F Fig). Nevertheless, even the reduced amount of PVA vRNA yielded a similar amount of RLUC as the control (S14E Fig). During VCS and VPg co-expression, even an eight-fold lower RNA concentration yielded the same amount of RLUC compared to the control (S14G and S14H Fig). Likewise in the case of replication-deficient PVA RNA VCS and VPg co-expression yielded similar RLUC expression from approximately 5.5 fold less RNA (S11A and S11B Fig). Our analysis did not show if the reduction in viral RNA occurred on polysomes during translation since we measured the total RNA amount. Therefore, more investigations are required to link this phenomenon to RNA silencing -related relief of translational repression.

PVA^WD^ infection did not produce detectable virus particles in spite of a substantial level of viral gene expression in the local leaves (Fig 8). Defective particle assembly process and particle instability may both explain why particles were not detected. Currently it is not possible to conclude which of these two alternatives is correct. In an earlier report we found that VCS knockdown affects the CP accumulation in PVA infection [10]. In the current this study with PVA^WD^ we also observed reduced CP accumulation compared to PVA^WT^ (Fig 8A). With expression of the PVA polyprotein, we have shown that RLUC activity from the 3’ side of PVA RNA is proportional to CP expression [49]. The reduced CP level in PVA^WD^ may also result from the degradation of free CP [50] due to defects in particle formation and gives further support for the role of HCPro-VCS interaction in virion formation. The absence of virions or the presence of unstable virions both could explain the low PVA^WD^ infection level in the systemic leaves. We tested this phenomenon also in a TuMV-*N*. *benthamiana* pathosystem [51]. As in PVA infection, the WD mutation in TuMV HCPro caused a defect in virion formation and impaired systemic movement. (S16 Fig). The complete absence of particles from PVA^WD^ -infected leaves when observed under transmission electron microscopy proposes that assembly of viable particles was not possible (Fig 8). Potyviral transmission between plants occurs when aphids absorb virus particles to their stylet during ingestion of leaf material. Therefore, especially the lack of particle stability in the absence of HCPro-VCS binding may have high practical relevance for viral resistance breeding.

Taken together, we propose that VCS is by nature an antiviral protein, which viral proteins HCPro and VPg have co-opted to promote PVA infection. The antiviral RNA silencing machinery targets viral RNA upon exit from viral replication complexes. We propose that HCPro recruits VCS, which serves as a scaffolding platform to assemble an RNP complex around viral RNA. This is a prerequisite not only for the protection of viral RNA from the host’s defence, but also for the production of viable PVA particles and long-distance transport of PVA.

## Methods

### Plants

All experiments were carried out using *Nicotiana benthamiana* plants as the host. The plants were grown in an environmentally controlled greenhouse at 18 h light and 6 h dark photoperiod at 22 ^o^C and 18 ^o^C respectively. Relative humidity was maintained at 50%.

### Recombinant viruses and HCPro overexpression constructs

Mutation in the WD40 domain-interacting motif of HCPro was introduced in the wild type P1-C-terminus-HCPro (or HCPro^Strep-RFP^) P3-N-terminus fragment (P1-HCPro-P3) in the pGEM-T-Easy vector (Promega) using a two-step site directed mutagenesis described by Edelheit et al. [52]. The primer sequences are as follows: forward primer- 5’-CAA GTG CCG CCG **C**GC TTC CA**G C**AA TTT TGG TTG-3’; reverse primer- 5’-CAA CCA AAA TT**G C**TG GAA GC**G** CGG CGG CAC TTG-3’ (changed nucleotides are marked in bold). The mutated fragments (P1-HCPro^WD^-P3 or P1-HCPro^WD-Strep-RFP^-P3) were then used to replace the P1-HCPro-P3 fragment in PVA or PVA^WT-Strep-RFP^ icDNAs in the pUC18 vector using SexAI and NruI restriction sites. A non-replicating variant PVA^ΔGDD-HCProWD^ was prepared by replacing the wild-type NIb gene with the mutated ΔGDD version as described in Eskelin et al. [29]. Finally, to enable *Agrobacterium* mediated transformation the mutated PVA icDNAs were transferred into the binary vector pRD400 using SalI and KpnI restriction sites.

For HCPro expression constructs, the mutation was introduced similarly in an interim vector containing 35S promoter-HCPro-*nos* terminator (pHTT690). The mutated 35S-HCPro^WD^-*nos* fragment was cloned into pRD400 using HindIII restriction sites. Expression constructs for TwinStrep-tagged RFP fusions of HCPro^WT^ and HCPro^WD^ were generated from P1-HCPro^WT-Strep-RFP^-P3 or P1-HCPro^WD-Strep-RFP^-P3 fragments by PCR using the following primers: forward- 5’-TCC GCT CGA GAT GAA ATG GTC TCA TCC ACA A-3’, reverse- 5’-CGC GGA TCC TCA CGA CTC TTT TTC GAA CTG-3’. The primers were designed to add an Xho1 restriction site at the 5’ end and both a stop codon and a BamH1 restriction site to the 3’ end of the amplified fragment. XhoI-BamHI digested PCR products were ligated into similarly double-digested pHTT690H to generate 35S-TwinStrep-RFP-HCPro-*nos* and 35S-TwinStrep-RFP-HCPro^WD^-*nos*. After sequence verification the expression cassettes were transferred into the pRD400 binary vector using HindIII restriction.

### *N*. *benthamiana* VARICOSE expression constructs

Genes homologous to the *Arabidopsis thaliana* VARICOSE (VCS, accession Q9LTT8) were identified with a BLAST search of the *N*. *benthamiana* genome v1.0.1 predicted cDNAs (https://solgenomics.net/). Three *N*. *benthamiana* VCS-like genes: Niben101Scf00654g02003.1 (VCS-A), Niben101Scf39216g00007.1 (VCS-B) and VCSC Niben101Scf21107g00015.1 (VCS-C) were Gateway-cloned into expression vectors. VCS-A and VCS-C were amplified with gateway-compatible primers from a *N*. *benthamiana* cDNA library generated from total RNA using Superscript III (Invitrogen). VCS-B was synthesized and cloned into the pUC57 vector by BaseClear, Netherlands. The forward primer for VCS-A and -C was 5’-GGG GAC AAG TTT GTA CAA AAA AGC AGG CTT CAT GGC TTT CTT TGC AGA G-3´ and the reverse primers were- 5’-GGG GAC CAC TTT GTA CAA GAA AGC TGG GTT TCA TTT ACT CAT CAA CAT CGA-3’ and 5’-GGG GAC CAC TTT GTA CAA GAA AGC TGG GTT TCA TTT ACT ACA GGT CAT CAG-3’, respectively. VCS-B was amplified with the forward primer 5’-GGG GAC AAG TTT GTA CAA AAA AGC AGG CTT CAT GGC TTC TTC TCC TGG C-3’ and the reverse primer 5’-GGG GAC CAC TTT GTA CAA GAA AGC TGG GTT TCA TTT ACT CAT CAA CAT CGA AT-3’. The PCR products were cloned into pDONR-Zeo via a BP-reaction with a BP ClonaseII enzyme mix (Invitrogen). Positive entry clones were verified by sequencing and LR-cloned into the KpnI-linearized destination vector pMDC32 to generate native expression constructs. XhoI-linearised pGWB42 [53] was used as the destination vector to construct N-terminal YFP-fusions.

### Agroinfiltration

Preparation of the infiltration mix as well as agroinfiltration was carried out following Ivanov et al. [12].

### Luciferase assay

Samples at designated dpi were collected using a cork borer of internal diameter 5-mm. Leaf discs were either directly used for luciferase assays using a Dual luciferase kit (Promega) as in Eskelin et al. [29] or stored at -80 ^o^C for later analysis.

### YFP Fluorescence quantification

YFP fluorescence intensity was quantified directly from 5-mm leaf discs using a Tecan Infinite M200 monochromator-based pate reader following De et al. [54]. Ex/Em wavelength used for the detection of YFP fluorescence was 500/530 nm.

### Epifluorescence and confocal microscopy

The expression and localization of HCPro^WD-Strep-RFP^, HCPro^WT-Strep-RFP^ and VCS^YFP^ in *N*. *benthamiana* epidermal cells was examined with confocal laser scanning microscopy using a Leica TCS SP5II instrument (Leica Microsystems). For imaging, leaf samples were mounted upside down on an objective slide with a drop of water and placed under a cover glass. All images were acquired with a 63x water immersion objective and in sequential scanning mode in order to avoid fluorophore bleed-through. YFP was excited with a 488 nm argon laser and RFP with a DPSS 561 nm laser. Emissions were detected at 525–555 nm and at 570–630 nm for YFP and RFP, respectively. CFP was excited with an argon laser at 458 nm and emission was recorded at 470–500 nm. Line-averaging was adjusted to 4 to improve image quality. Co-localization analysis of VCS^YFP^ with HCPro^WT-Strep-RFP^ and HCPro^WD-Strep-RFP^ was performed with the Fiji (ImageJ) image analysis software package using the co-localization threshold-function. HCPro-containing granules were selected as ROIs for the co-localization and results are given as % intensity of VCS^YFP^ co-localizing with HCPro^WT-Strep-RFP^ or HCPro^WD-Strep-RFP^.

PG visualization and quantification was carried out using epifluorescence microscopy following the method described in Hafrén et al. [10].

### Strep-Tag affinity purification of HCPro and identification of co-purified proteins

HCPro-associated protein complexes were isolated from leaves infected with PVA^WT-Strep-RFP^ or PVA^WD-Strep-RFP^ infiltrated at OD_600_ = 0.1. GUS-infiltrated plants were used as negative controls. Leaves were harvested at 3 dpi and immediately frozen in liquid nitrogen. Subsequently, Strep-tag mediated purification of HCPro^WT-Strep-RFP^ or HCPro^WD-Strep-RFP^ was carried out following Ivanov et al. [30] with minor modifications. 1 g frozen leaf material was homogenized in 3 mL binding buffer. The homogenate was subsequently filtered through double layered miracloth and centrifuged at 5000 ×g for 5 min at 4 ^o^C to remove insoluble debris. Approximately 2 mL of cleared lysate was mixed with 50 μL resin suspension and incubated overnight with gentle rotation at 4 ^o^C. The rest of the protocol is same as Ivanov et al. [30]. Final elution volume was 100 μL. Identification of proteins present in the purified fractions was carried out by western blotting and LC-MS/MS following Ivanov et al. [12].

### DNase, RNase and Proteinase K stability assay

Purified products obtained after elution from Strep-Tactin resin were subjected to DNase (RQ1 RNase-Free DNase, Promega), Proteinase K (Product No. 59895, Invitrogen, Thermo Fisher Scientific) and RNase A (Fermentas) treatment. Each reaction mix comprised 3 μL eluate and 1 μL DNase (1u/μL), Proteinase K (20 μg/μL) or RNase A (10 μg/μL). Final reaction volume was made up to 10 μL with recommended buffer / water. Reaction mixes were incubated for 1 h at 37 ^o^C and thereafter subjected to standard SDS-PAGE followed by silver staining.

### Western blotting

Standard western blotting procedures were followed to detect eluted proteins from Strep-affinity purification experiments and from all other overexpression experiments. Western blot for HCPro was carried out following De et al. [54], while SAMS, AGO1, CI and VPg were detected similarly to Ivanov et al. [12]. A customised anti-VCS antibody recognizing all three *N*. *benthamiana* VCS proteins used in this study was produced in rabbit (Biomatik) and used at 1 μg/mL dilution. The antibody recognizes peptide sequence ‘TEGPDEEDKPQITGK’ located near the N-terminal region of all three forms of VCS (S17 Fig).

### LC-MS/MS

LC-MS/MS analysis of the purified products were carried out following Ivanov et al. [12].

### RT and ic-qRT-PCR

Omega E.Z.N.A. Plant RNA-purification kit (Product No. R6827-02) along with RNase-free DNase (Product No. E1091-02) was used to purify RNA from 100 mg of leaf tissues. Standard protocol [55] for ic-qRT-PCR was followed to quantify PVA particles from 100 mg of leaf tissue. Primary antibody against CP (SASA, UK) at 1:1000 dilution was used for this purpose. All cDNAs were prepared using the H Minus First Strand cDNA synthesis kit (Product No. K1632, Thermo Fisher Scientific) with random hexamer primers. qPCR was carried out using Maxima SYBR Green qPCR Master mix (Thermo Fisher Scientific) and a CFX96 Touch^TM^ Real-Time PCR Detection System. Amplification of PVA RNA was carried out using primers targeting the CP region: forward- 5’-CAT GCC CAG GTA TGG TCT TC-3’, reverse- 5’-ATC GGA GTG GTT GCA GTG AT-3’. The protein phosphatase 2A (PP2A) gene was used as the reference gene. PP2A was amplified with the following primers: forward- 5’-GAC CCT GAT GTT GAT GTT CG-3’, reverse- 5’-GAG GGA TTT GAA GAG AGA TTT C-3’.

### Electron microscopy

PVA particles were visualized with a Jeol JEM-1400 transmission electron microscope (Jeol Ltd., Tokyo, Japan). 100 mg leaf tissue was ground in 100 μL 0.06 M phosphate buffer and incubated on ice for 1 h to let the cell debris settle. Carbon-coated EM-grids were incubated with the anti-CP antibody (1:100 dilution) for 1 h at room temperature. Excess antibody was washed with phosphate buffer. Subsequently, the antibody-coated grids were incubated for 5 min with supernatant collected from the leaf extracts after settling down of the debris. Grids were further washed with 20 drops of phosphate buffer and immediately stained with 2% Uranyl acetate for 15 sec. Excess stain was drained from the grids using filter paper and dried grids were used for visualization of the particles. EM images of the infected leaf tissues were visualized following Lõhmus et al. [56].

## Supporting information

S1 Fig WD interacting domains in HCPro- conservation and characterization. Identification of motifs within the HCPro sequence with potential to interact with WD domain proteins (A) 119 potyviral HCPro amino acid sequences were aligned and searched for interaction motifs for WD domain proteins. Six highly conserved, putative WD domain -interacting motifs were found. These sequences and the percentage of their conservation among potyviruses are indicated in (A). ‘C’ and ‘H’ residues that are important for cysteine protease activity of HCPro are marked with arrow in the alignment shown in (A). Variation in the amino acids within each motif is presented in the table below. Motif no. 5, the AELPR sequence used in this study, is the most conserved one. (B) Localization of the motifs 3-5 within the crystal structure of the C-terminal domain of TuMV HCPro (PDB id-3RNV). Motifs 1 and 2 are outside the resolved structure and motif 6 is not conserved in TuMV HCPro. Hence, they could not be shown. Motifs 3 and 4 were merged and are highlighted with yellow whereas motif 5 is indicated with red. (C, D) Ribbon diagrams of VCS proteins from Arabidopsis thaliana, obtained from WDSP database (http://www.wdspdb.com/wdsp/). (C) AT3G13300.1 and (D) AT3G13300.3. (E) Modelling the molecular docking of HCPro (from (B)) and VCS (from (C)) was carried out using ClusPro online server (https://cluspro.bu.edu/). Similarly, (F) presents the predicted interaction model between (B) and (D). The docking model in which the AELPR motif interacts with VCS was among the top predictions.
(TIF)

S2 Fig Expression levels of HCPro^WT-Strep-RFP^ / HCPro^WD-Strep-RFP^ expression constructs following *Agrobacterium* infiltration at different ODs. HCPro^WD-Strep-RFP^ / HCPro^WT-Strep-RFP^ constructs were *Agrobacterium* infiltrated at different ODs (OD_600_ = 0.01; 0.05; 0.1; 0.5). The RFP fluorescence level at Ex/Em = 555/584 nm was measured from the intact leaf discs with a microplate reader. Statistically significant differences between the samples are denoted by asterisks (**P < 0.01; ***P < 0.001; *n = 6*). In spite of the statistically significant differences in the HCPro^WD-Strep-RFP^ / HCPro^WT-Strep-RFP^ accumulation levels, they did not differ drastically.
(TIF)

S3 Fig Control images showing the absence of granules in P0^YFP^ overexpression alone. Control images showing the absence of PG formation by P0^YFP^ overexpression alone (ref. Fig 3E and 3F). GUS is used to balance the *Agrobacterium* cell count in the absence of HCPro.
(TIF)

S4 Fig Pairwise overlays: HCPro^WT-Strep-RFP^ / HCPro^WD-Strep-RFP^ and VCS^YFP^; HCPro^WD-Strep-RFP^ / HCPro^WT-Strep-RFP^ and P0^CFP^. Presented are the pairwise overlays between HCPro^WT-Strep-RFP^ (A-C) / HCPro^WD-Strep-RFP^ (G-I) and VCS^YFP^ and HCPro^WT-Strep-RFP^ (D-F) / HCPro^WD-Strep-RFP^ (J-L) and P0^CFP^ to support the result presented in Fig 4A–4F.
(TIF)

S5 Fig Expression levels of HCPro^WT-Strep-RFP^, HCPro^WD-Strep-RFP^ and VCS^YFP^ in the experiment presented in (Fig 4A–4F). RFP and YFP fluorescence levels in the intact leaf discs measured at Ex/Em 555/584 nm and 500/530 nm, respectively, in a microplate reader. Statistically significant differences between the samples are denoted by an asterisk (**P < 0.01); *n = 6*
(TIF)

S6 Fig Degree of co-localization between HCPro and VCS corresponding to experiment presented in (Fig 4G–4L). HCPro-containing foci (= PGs) were selected as region of interests (ROIs) for the co-localization and the results are given in terms of % (by intensity) of VCS^YFP^ co-localizing with HCPro^WD-Strep-RFP^ / HCPro^WT-Strep-RFP^. Statistical significance was calculated from images taken from three independent sets of biological replicates (*n = 11*; student's t-test ***P < 0.001).
(TIF)

S7 Fig Relative expression levels of HCPro^WT-Strep-RFP^ / HCPro^WD-Strep-RFP^ and VCS^YFP^ in Fig 4G–4L. RFP fluorescence was measured at Ex/Em = 555/584 nm while YFP fluorescence was measured at Ex/Em = 500/530 nm from the intact leaf discs using a microplate reader. Statistically significant differences between the samples are denoted by asterisks (**P < 0.01; N.S. stands for non-significant; *n* = 48).
(TIF)

S8 Fig Epifluorescence microscopy images showing that HCPro^WD-Strep-RFP^ aggregates and PGs visualized by P0^YFP^ marker are distinct entities. (A) Percentage of HCPro aggregates co-localizing with P0^YFP^. Significance of the differences between the samples is denoted by asterisk (***P < 0.001; *n* = 5). (B-D) HCPro^WD-Strep-RFP^ aggregates, P0^YFP^ and their overlay within a single cell under a 100X water immersion objective. (E-G) Reference PGs with HCPro^WT-Strep-RFP^ and P0^YFP^ shows co-localization. Samples were visualized at 3 dpi. Experimental details are the same as in Fig 4M–4R. HCPro and P0^YFP^ were visualized at 3 dpi with an epifluorescence microscope using RFP and YFP filters respectively.
(TIF)

S9 Fig Validation of enrichment of HCPro-associated HMW complexes during Twin-Strep-tag affinity purification in samples collected from PVA^WT-Strep-RFP^ / PVA^WD-Strep-RFP^ infected local leaves at 3 dpi. Both inputs and eluates containing equivalent amount of total protein (6 μg total protein measured by using Qubit™ Protein Assay Kit), were loaded as indicated. Left panel shows the silver-stained gel of the inputs and eluates while the right panel shows the western blots of the same samples as investigated with anti-HCPro and anti-VCS antibodies (A). While the anti-HCPro western blot indicates strong enrichment of HCPro-containing HMW complexes, the anti-VCS western blot confirms the absence of the VCS from the uppermost HMW band from PVA^WD-Strep-RFP^ purified fraction (marked with arrow). Monomeric HCPro was also detected by the anti-HCPro antibody (marked with asterisk). Neither HCPro nor VCS could be detected from the input samples in the western blots. Therefore their presence in the inputs was studied by loading 40-times higher total protein amounts than in S9A Fig. (B) Both HCPro and VCS were detected faintly in the respective input blots.
(TIF)

S10 Fig Validation of VCS silencing presented in Fig 6A–6C. Validation of VCS silencing via semi-quantitative RT-PCR. Panel (A) corresponds to the sample sets shown in Fig 6A and 6B while Panel (B) corresponds to those presented in Fig 6C and 6D. Validation of VCS mRNA silencing was done by comparing band intensities in the agarose gel. Housekeeping gene PP2A served as a loading control. Both VCS and PP2A fragments were amplified for 27 PCR cycles.
(TIF)

S11 Fig RLUC expression and RNA accumulation levels used to calculate RLUC/RNA ratio presented in Fig 7A and validation of VCS overexpression. (A) Amount of RLUC activity detected from non-replicating variants of PVA, PVA- PVA^ΔGDD-ΔHCPro^, PVA^ΔGDD-HCProWD^ and PVA^ΔGDD-HCProWT^ during VPg, VCS and VPg + VCS overexpression (B) Amount of RNA accumulation detected from non-replicating variants of PVA- PVA^ΔGDD-ΔHCPro^, PVA^ΔGDD-HCProWD^ and PVA^ΔGDD-HCProWT^ during VPg, VCS and VPg + VCS overexpression (C,D) Validation of VCS overexpression during the experiments discussed in Fig 7A. For confirmation of VCS overexpression qPCRs were carried out with primers specific to VCS-A, VCS-B and VCS-C respectively. Different letters above the bars indicate statistically significant differences (student's t-test *P* < 0.05). Significance of the differences between the compared samples is denoted by asterisk (*P < 0.05, **P < 0.01, ***P < 0.001).
(TIF)

S12 Fig Complementation of VPg- mediated translational enhancement of PVA^ΔGDD-ΔHCPro^ by various HCPro mutants. *Agrobacterium* carrying different HCPro variants were infiltrated as follows: silencing-deficient HCPro^SDM^ and eIF4E binding-deficient HCPro^4EBM^ mutants at OD_600_ = 1, HCPro^WD^ and HCPro^WT^ at OD_600_ = 0.3. GUS was used as the control, as well as to balance *Agrobacterium* counts between the sets. PVA^ΔGDD-ΔHCPro^ was infiltrated at OD_600_ = 0.05 and VPg infiltrated at OD_600_ = 0.3. Samples for RLUC quantitation were collected at 3 dpi. The number of plants per experiment was 6. Different letters above the bars indicate statistically significant differences (student's t-test *P* < 0.05).
(TIF)

S13 Fig Validation of VCS silencing and overexpression during the experiments discussed in Fig 7. (A, B) For validation of VCS silencing in Fig 7B and 7C, VCS mRNA levels were detected by qPCR using primer pair recognizing all three forms of VCS. (C, D) For confirmation of VCS overexpression in Fig 7D and 7E, qPCRs were carried out with primers specific to VCS-A, VCS-B and VCS-C respectively. Significance of the differences between the compared samples is denoted by asterisk (*P < 0.05, **P < 0.01, ***P < 0.001).
(TIF)

S14 Fig RLUC expression and RNA accumulation levels used for calculating RLUC/RNA ratio presented in Fig 7. (A) Amount of RLUC activity detected from replicating variants of PVA- PVA^ΔHCPro^, PVA^WD^ and PVA^WT^ upon VCS-silencing (B) Amount of RNA accumulation detected from replicating variants of PVA- PVA^ΔHCPro^, PVA^WD^ and PVA^WT^ upon VCS-silencing (C) Amount of RLUC activity detected from replicating variants of PVA- PVA^ΔHCPro^, PVA^WD^ and PVA^WT^ upon VCS-silencing and VPg overexpression (D) Amount of RNA accumulation detected from replicating variants of PVA- PVA^ΔHCPro^, PVA^WD^ and PVA^WT^ upon VCS-silencing and VPg overexpression (E) Amount of RLUC activity detected from replicating variants of PVA- PVA^ΔHCPro^, PVA^WD^ and PVA^WT^ upon VCS overexpression (F) Amount of RNA accumulation detected from replicating variants of PVA- PVA^ΔHCPro^, PVA^WD^ and PVA^WT^ upon VCS overexpression (G) Amount of RLUC activity detected from replicating variants of PVA- PVA^ΔHCPro^, PVA^WD^ and PVA^WT^ upon VCS and VPg overexpression (H) Amount of RNA accumulation detected from replicating variants of PVA- PVA^ΔHCPro^, PVA^WD^ and PVA^WT^ upon VCS and VPg overexpression. Significance of the differences between the compared samples is denoted by asterisk (*P < 0.05, **P < 0.01).
(TIF)

S15 Fig Both HCPro^WD^ and HCPro^WT^ are associated with polysomes during infection. Polysomes were purified from PVA^WD-Strep-RFP^ (OD_600_ = 0.3) and PVA^WT-Strep-RFP^ (OD_600_ = 0.1) infected *N*. *benthamiana* plants (sampled at 4 dpi), via a Assymetric Field Flow Fractionation (AF4) protocol previously described in Eskelin et al. (2019). (A) Upper panel presents AF4 elution profiles of representative PVA^WD-Strep-RFP^ infected samples while the lower panel presents those of representative PVA^WT-Strep-RFP^ infected samples. Polysome fraction from the eluate (between 15–70 min) were pooled together and concentrated using a 10kDa MW cutoff centrifugation filter (Amicon). (B) Concentrated eluates were subjected to SDS-PAGE and the gels were silver stained (upper panel) and subjected to western blotting with anti-HCPro antibody (lower panel). Silver stained gel images for concentrated eluates show clear enrichment of ribosomal proteins (marked in the image between 55-25 kDa). The western blot revealed signals corresponding to both HCPro^WD^ and HCPro^WT^ both in the monomeric (marked by ‘*’) and HMW region (marked by ‘**’). Interestingly, band for HCPro^WD-Strep-RFP^ at the HMW region was visibly lower than that of the HCPro^WT-Strep-RFP^.
(TIF)

S16 Fig TuMV systemic infection and particle formation are affected by mutation in the WD-interacting motif of HCPro. Systemic infection and particle abundance are reduced in TuMV^WD^ compared to the control virus TuMV^WT^. Student’s t-test was used to calculate satistical significance (Significance of the differences between the compared samples is denoted by asterisk (*P < 0.05, **P < 0.01). (A) Abundance of TuMV CP in systemically infected *N*. *benthamiana* at 14 dpi. Plants were infiltrated by TuMV^WT^ and TuMV^WD^ (OD_600_ = 0.5) and samples from systemic leaves were analysed by anti-TuMV CP ELISA (Agdia). (B) Relative TuMV particle abundance in systemically infected *N*. *benthamiana* leaves at 12 dpi (OD_600_ = 0.5). Particle abundance was measured by IC-RT-PCR.
(TIF)

S17 Fig α-VCS western blot showing that all three forms of VCS are recognized by the antibody. α-VCS western blot showing that all three forms of VCS are recognized by the anti-VCS antibody. For this experiment VCS-A^YFP^, VCS-B^YFP^ and VCS-C^YFP^ were overexpressed independently in N. benthamiana (infiltrated at OD_600_ 0.5). Samples were collected at 3 dpi followed by affinity purification by GFP trap (ChromoTek). The eluates were analyzed by SDS-PAGE and anti-VCS western blot (antibody dilution 1 μg/ml).
(TIF)

S1 Table Recombinant constructs used in the study. (DOCX)

S2 Table LC-MS/MS identification of proteins present in HMW complexes produced by HCPro^WT-Strep-RFP^ but not in HCPro^WD-Strep-RFP^. (DOCX)

S1 Data Excel spreadsheet containing, in separate sheets, the underlying numerical data and statistical analysis for Figure panels 2A, 2B, 2C, 3B, 3C, 3D, 3G, 6A, 6B, 6C, 6D, 7A, 7B, 7C, 7D, 7E and 8A. (XLSX)

S1 Reference References related to the S1 Table and S15 Fig. (DOCX)
