# Supplementary figures and images for: Association of host protein VARICOSE with HCPro within a multiprotein complex is crucial for RNA silencing suppression, translation, encapsidation and systemic spread of potato virus A infection
Source: PLoS Pathog. 2020 Oct 12;16(10):e1008956. doi: 10.1371/journal.ppat.1008956 (PMC7581364; doi:10.1371/journal.ppat.1008956)

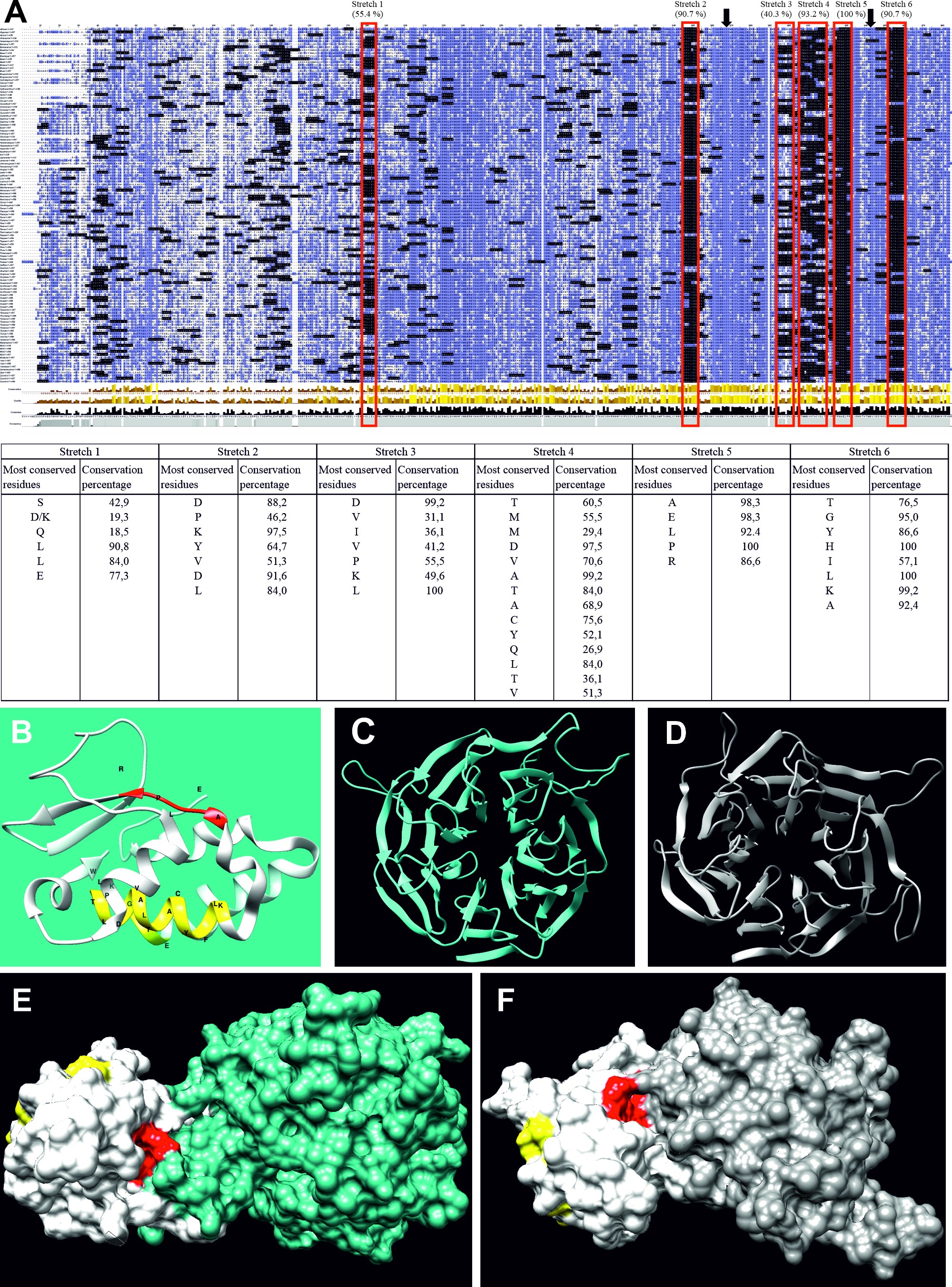

Supplement: S1 Fig — Identification of motifs within the HCPro sequence with potential to interact with WD domain proteins (A) 119 potyviral HCPro amino acid sequences were aligned and searched for interaction motifs for WD domain proteins. Six highly conserved, putative WD domain -interacting motifs were found. These sequences and the percentage of their conservation among potyviruses are indicated in (A). ‘C’ and ‘H’ residues that are important for cysteine protease activity of HCPro are marked with arrow in the alignment shown in (A). Variation in the amino acids within each motif is presented in the table below. Motif no. 5, the AELPR sequence used in this study, is the most conserved one. (B) Localization of the motifs 3-5 within the crystal structure of the C-terminal domain of TuMV HCPro (PDB id-3RNV). Motifs 1 and 2 are outside the resolved structure and motif 6 is not conserved in TuMV HCPro. Hence, they could not be shown. Motifs 3 and 4 were merged and are highlighted with yellow whereas motif 5 is indicated with red. (C, D) Ribbon diagrams of VCS proteins from Arabidopsis thaliana, obtained from WDSP database (http://www.wdspdb.com/wdsp/). (C) AT3G13300.1 and (D) AT3G13300.3. (E) Modelling the molecular docking of HCPro (from (B)) and VCS (from (C)) was carried out using ClusPro online server (https://cluspro.bu.edu/). Similarly, (F) presents the predicted interaction model between (B) and (D). The docking model in which the AELPR motif interacts with VCS was among the top predictions. (TIF) [file ppat.1008956.s001.tif]

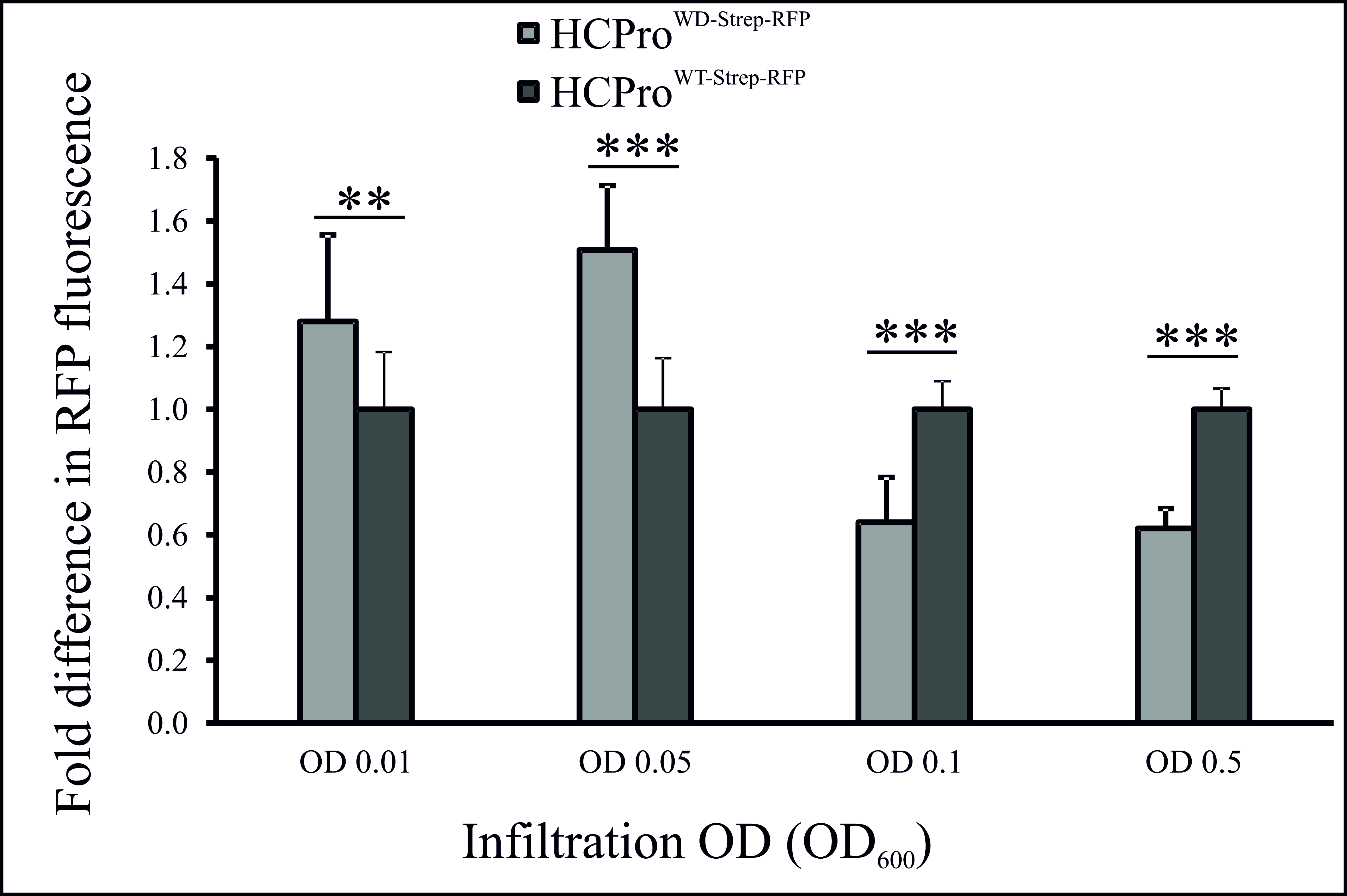

Supplement: S2 Fig — HCProWD-Strep-RFP / HCProWT-Strep-RFP constructs were Agrobacterium infiltrated at different ODs (OD600 = 0.01; 0.05; 0.1; 0.5). The RFP fluorescence level at Ex/Em = 555/584 nm was measured from the intact leaf discs with a microplate reader. Statistically significant differences between the samples are denoted by asterisks (**P < 0.01; ***P < 0.001; n = 6). In spite of the statistically significant differences in the HCProWD-Strep-RFP / HCProWT-Strep-RFP accumulation levels, they did not differ drastically. (TIF) [file ppat.1008956.s002.tif]

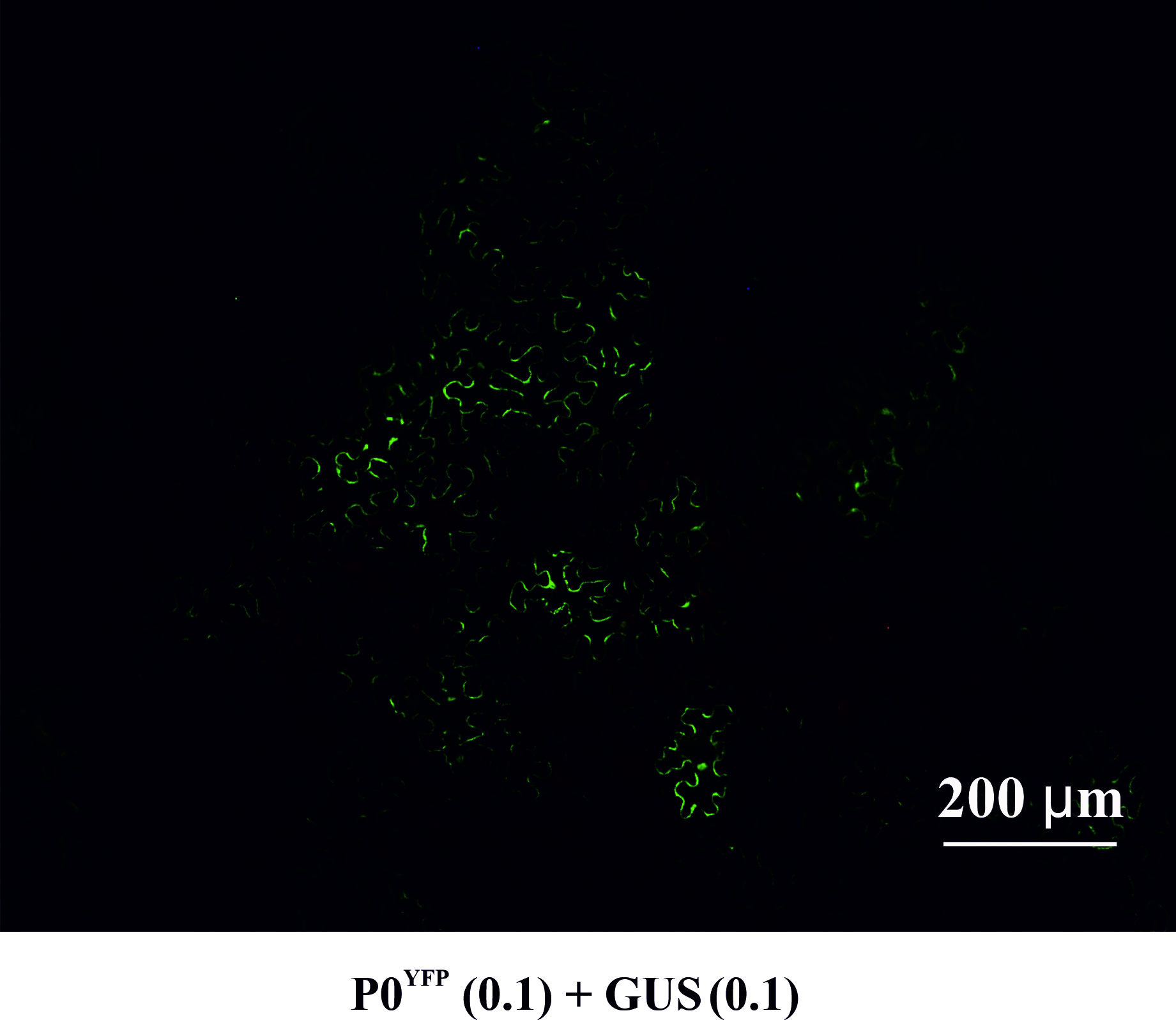

Supplement: S3 Fig — Control images showing the absence of PG formation by P0YFP overexpression alone (ref. Fig 3E and 3F). GUS is used to balance the Agrobacterium cell count in the absence of HCPro. (TIF) [file ppat.1008956.s003.tif]

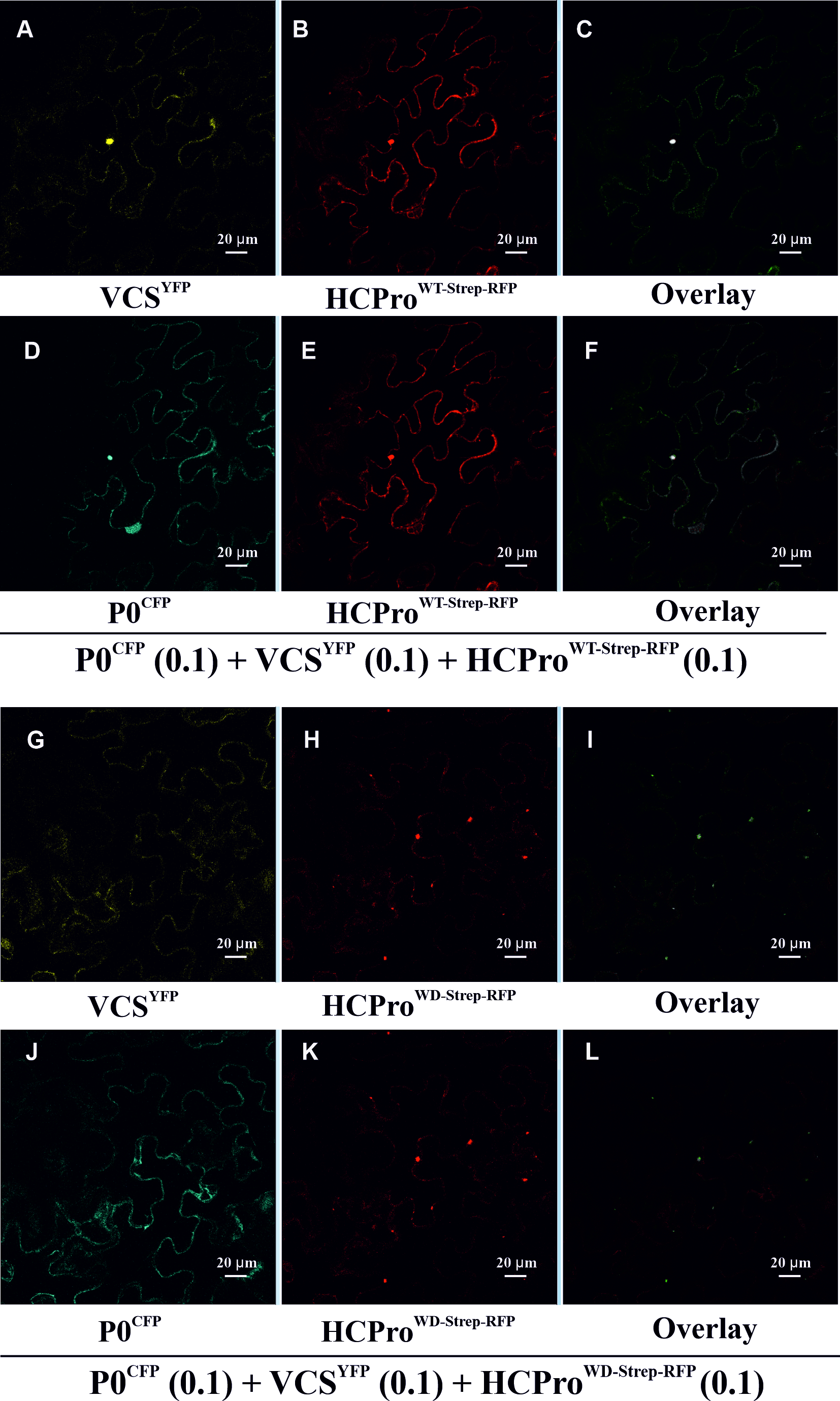

Supplement: S4 Fig — Presented are the pairwise overlays between HCProWT-Strep-RFP (A-C) / HCProWD-Strep-RFP (G-I) and VCSYFP and HCProWT-Strep-RFP (D-F) / HCProWD-Strep-RFP (J-L) and P0CFP to support the result presented in Fig 4A–4F. (TIF) [file ppat.1008956.s004.tif]

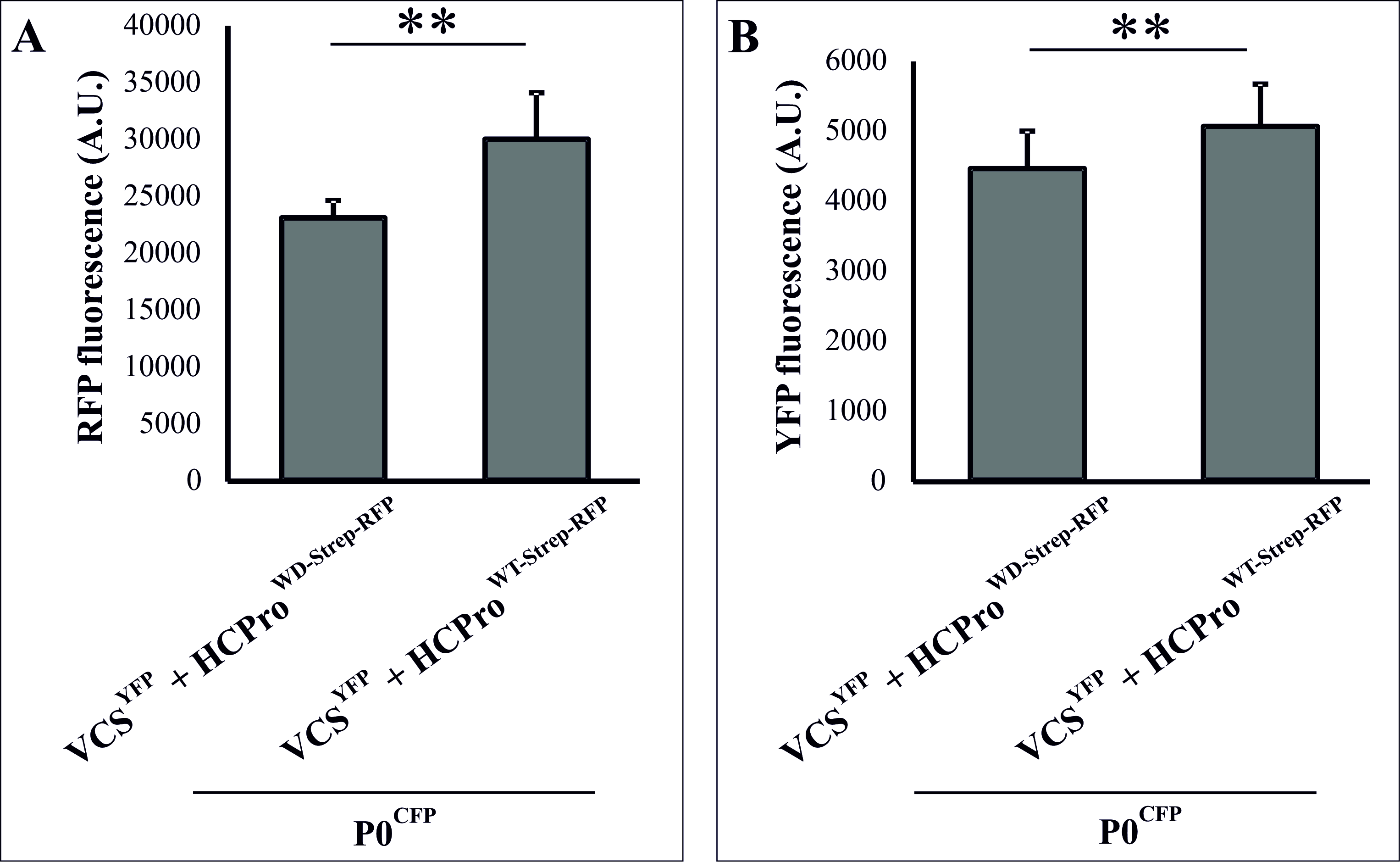

Supplement: S5 Fig — RFP and YFP fluorescence levels in the intact leaf discs measured at Ex/Em 555/584 nm and 500/530 nm, respectively, in a microplate reader. Statistically significant differences between the samples are denoted by an asterisk (**P < 0.01); n = 6 (TIF) [file ppat.1008956.s005.tif]

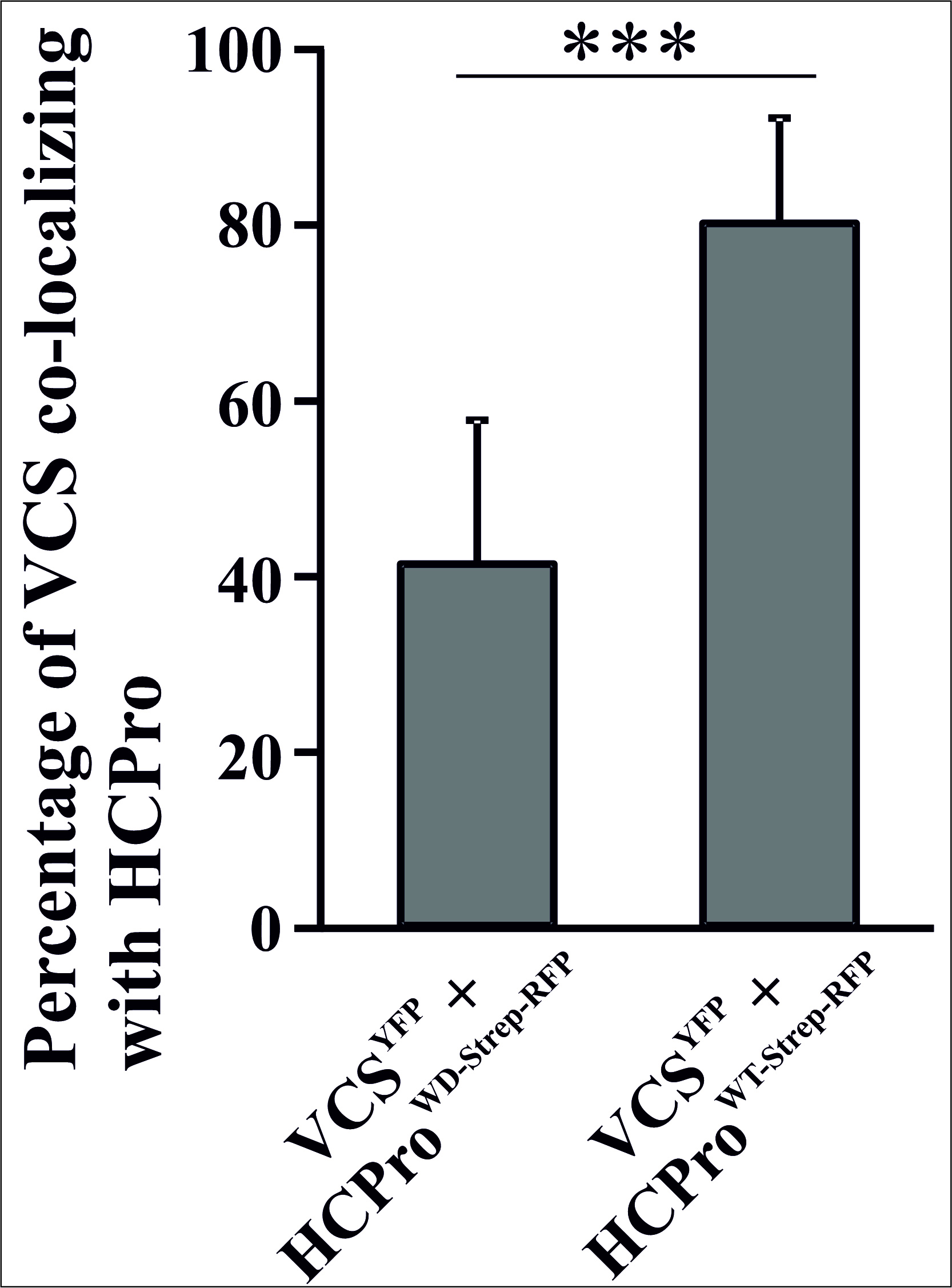

Supplement: S6 Fig — HCPro-containing foci (= PGs) were selected as region of interests (ROIs) for the co-localization and the results are given in terms of % (by intensity) of VCSYFP co-localizing with HCProWD-Strep-RFP / HCProWT-Strep-RFP. Statistical significance was calculated from images taken from three independent sets of biological replicates (n = 11; student's t-test ***P < 0.001). (TIF) [file ppat.1008956.s006.tif]

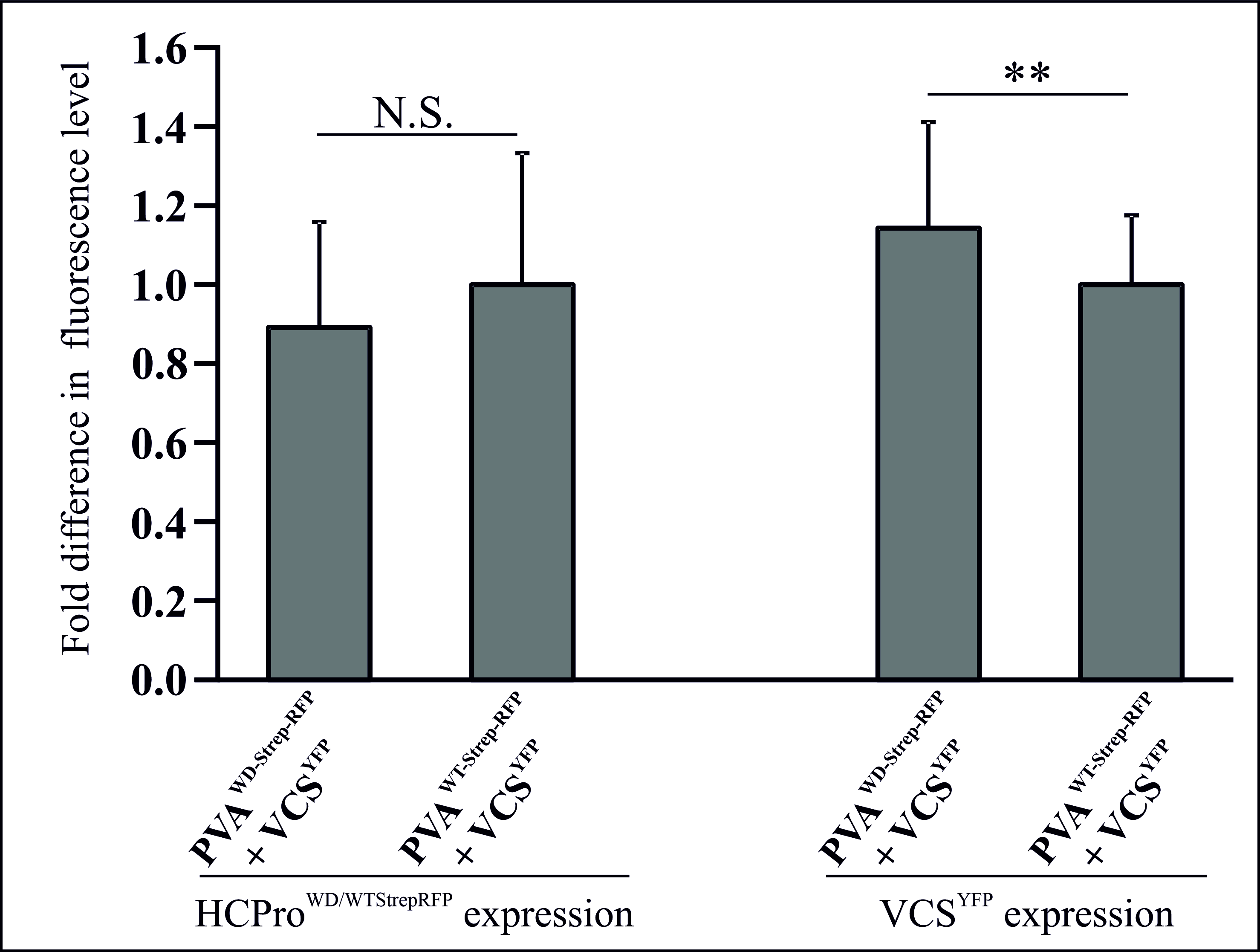

Supplement: S7 Fig — RFP fluorescence was measured at Ex/Em = 555/584 nm while YFP fluorescence was measured at Ex/Em = 500/530 nm from the intact leaf discs using a microplate reader. Statistically significant differences between the samples are denoted by asterisks (**P < 0.01; N.S. stands for non-significant; n = 48). (TIF) [file ppat.1008956.s007.tif]

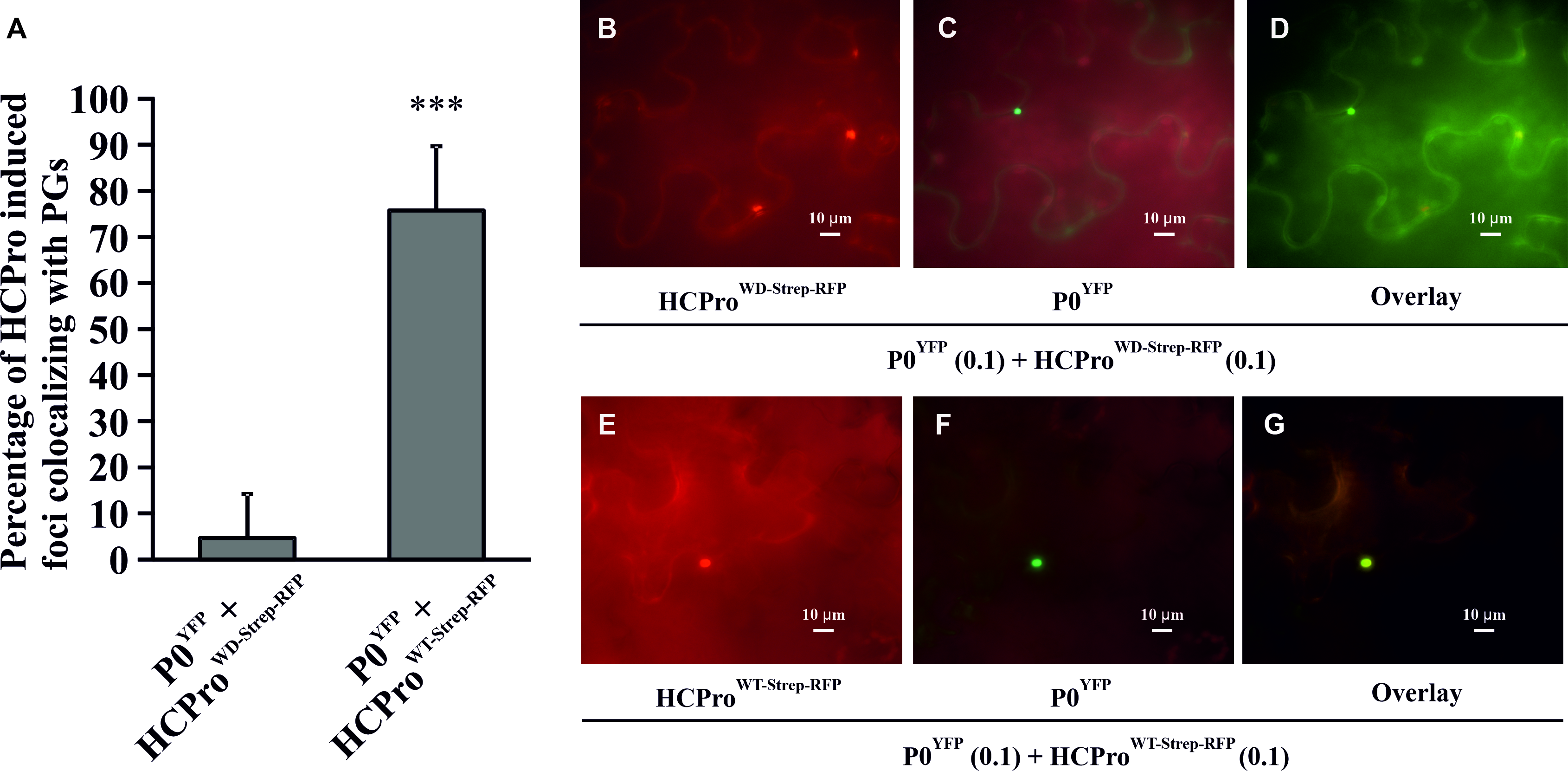

Supplement: S8 Fig — (A) Percentage of HCPro aggregates co-localizing with P0YFP. Significance of the differences between the samples is denoted by asterisk (***P < 0.001; n = 5). (B-D) HCProWD-Strep-RFP aggregates, P0YFP and their overlay within a single cell under a 100X water immersion objective. (E-G) Reference PGs with HCProWT-Strep-RFP and P0YFP shows co-localization. Samples were visualized at 3 dpi. Experimental details are the same as in Fig 4M–4R. HCPro and P0YFP were visualized at 3 dpi with an epifluorescence microscope using RFP and YFP filters respectively. (TIF) [file ppat.1008956.s008.tif]

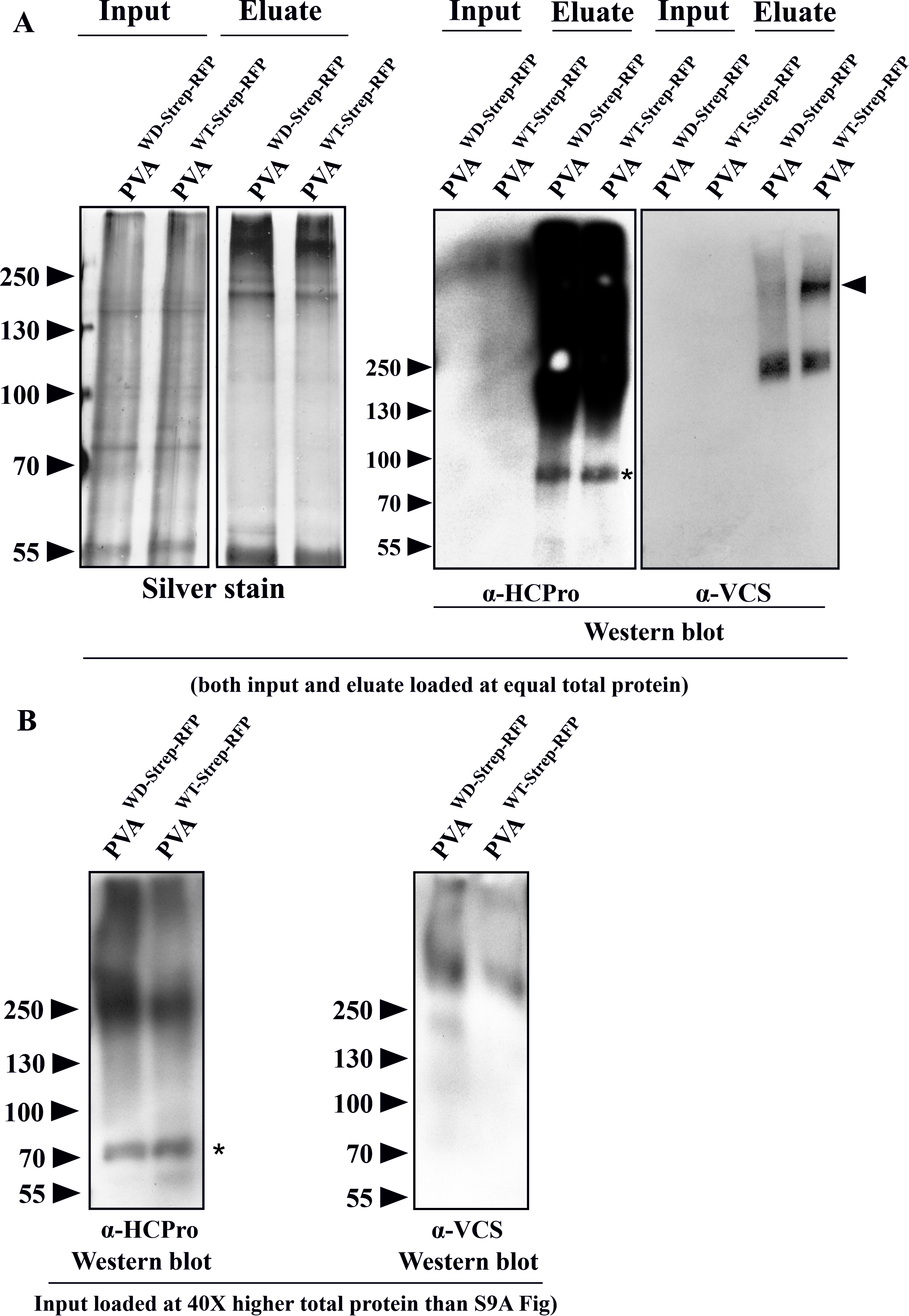

Supplement: S9 Fig — Both inputs and eluates containing equivalent amount of total protein (6 μg total protein measured by using Qubit™ Protein Assay Kit), were loaded as indicated. Left panel shows the silver-stained gel of the inputs and eluates while the right panel shows the western blots of the same samples as investigated with anti-HCPro and anti-VCS antibodies (A). While the anti-HCPro western blot indicates strong enrichment of HCPro-containing HMW complexes, the anti-VCS western blot confirms the absence of the VCS from the uppermost HMW band from PVAWD-Strep-RFP purified fraction (marked with arrow). Monomeric HCPro was also detected by the anti-HCPro antibody (marked with asterisk). Neither HCPro nor VCS could be detected from the input samples in the western blots. Therefore their presence in the inputs was studied by loading 40-times higher total protein amounts than in S9A Fig. (B) Both HCPro and VCS were detected faintly in the respective input blots. (TIF) [file ppat.1008956.s009.tif]

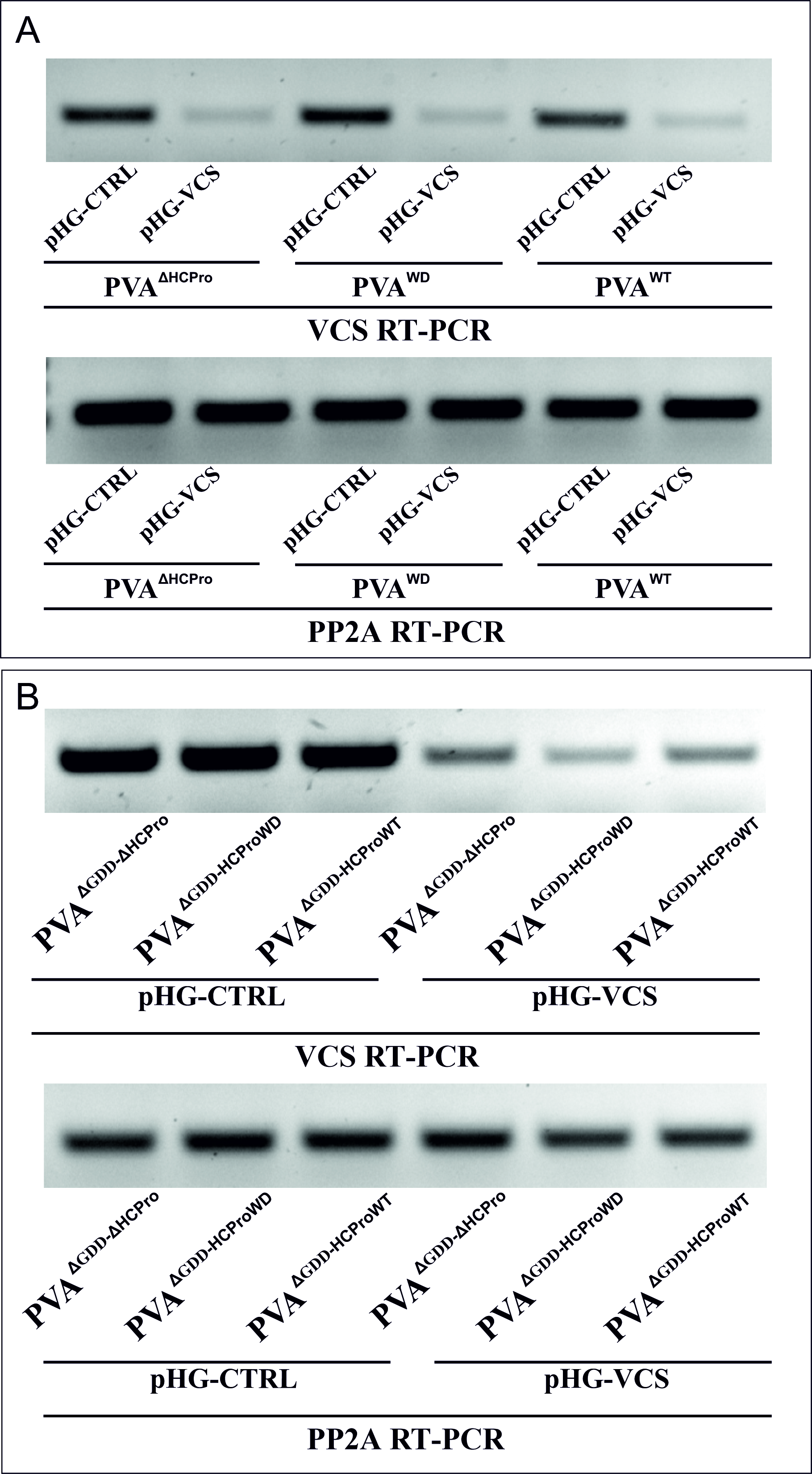

Supplement: S10 Fig — Validation of VCS silencing via semi-quantitative RT-PCR. Panel (A) corresponds to the sample sets shown in Fig 6A and 6B while Panel (B) corresponds to those presented in Fig 6C and 6D. Validation of VCS mRNA silencing was done by comparing band intensities in the agarose gel. Housekeeping gene PP2A served as a loading control. Both VCS and PP2A fragments were amplified for 27 PCR cycles. (TIF) [file ppat.1008956.s010.tif]

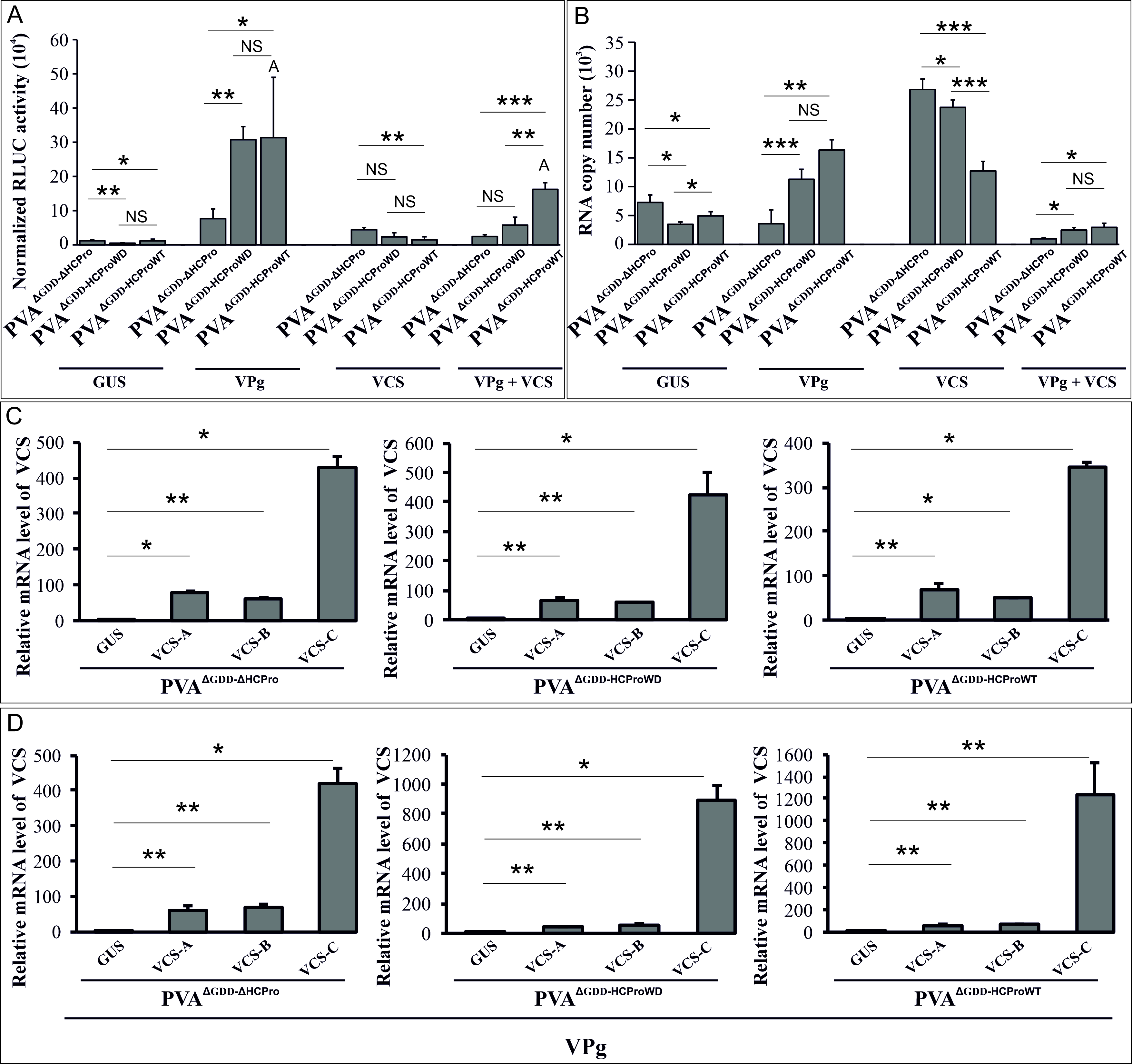

Supplement: S11 Fig — (A) Amount of RLUC activity detected from non-replicating variants of PVA, PVA- PVAΔGDD-ΔHCPro, PVAΔGDD-HCProWD and PVAΔGDD-HCProWT during VPg, VCS and VPg + VCS overexpression (B) Amount of RNA accumulation detected from non-replicating variants of PVA- PVAΔGDD-ΔHCPro, PVAΔGDD-HCProWD and PVAΔGDD-HCProWT during VPg, VCS and VPg + VCS overexpression (C,D) Validation of VCS overexpression during the experiments discussed in Fig 7A. For confirmation of VCS overexpression qPCRs were carried out with primers specific to VCS-A, VCS-B and VCS-C respectively. Different letters above the bars indicate statistically significant differences (student's t-test P < 0.05). Significance of the differences between the compared samples is denoted by asterisk (*P < 0.05, **P < 0.01, ***P < 0.001). (TIF) [file ppat.1008956.s011.tif]

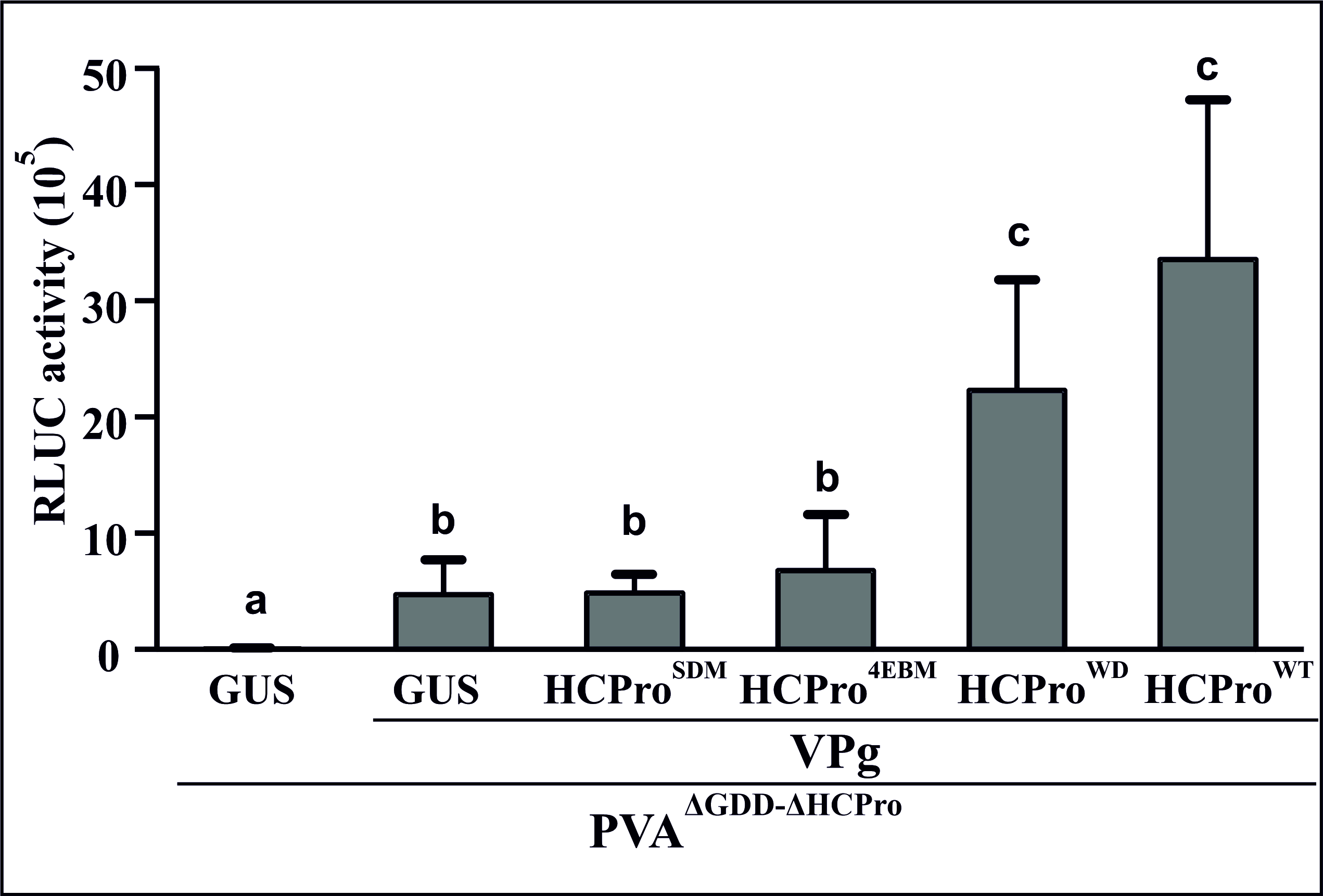

Supplement: S12 Fig — Agrobacterium carrying different HCPro variants were infiltrated as follows: silencing-deficient HCProSDM and eIF4E binding-deficient HCPro4EBM mutants at OD600 = 1, HCProWD and HCProWT at OD600 = 0.3. GUS was used as the control, as well as to balance Agrobacterium counts between the sets. PVAΔGDD-ΔHCPro was infiltrated at OD600 = 0.05 and VPg infiltrated at OD600 = 0.3. Samples for RLUC quantitation were collected at 3 dpi. The number of plants per experiment was 6. Different letters above the bars indicate statistically significant differences (student's t-test P < 0.05). (TIF) [file ppat.1008956.s012.tif]

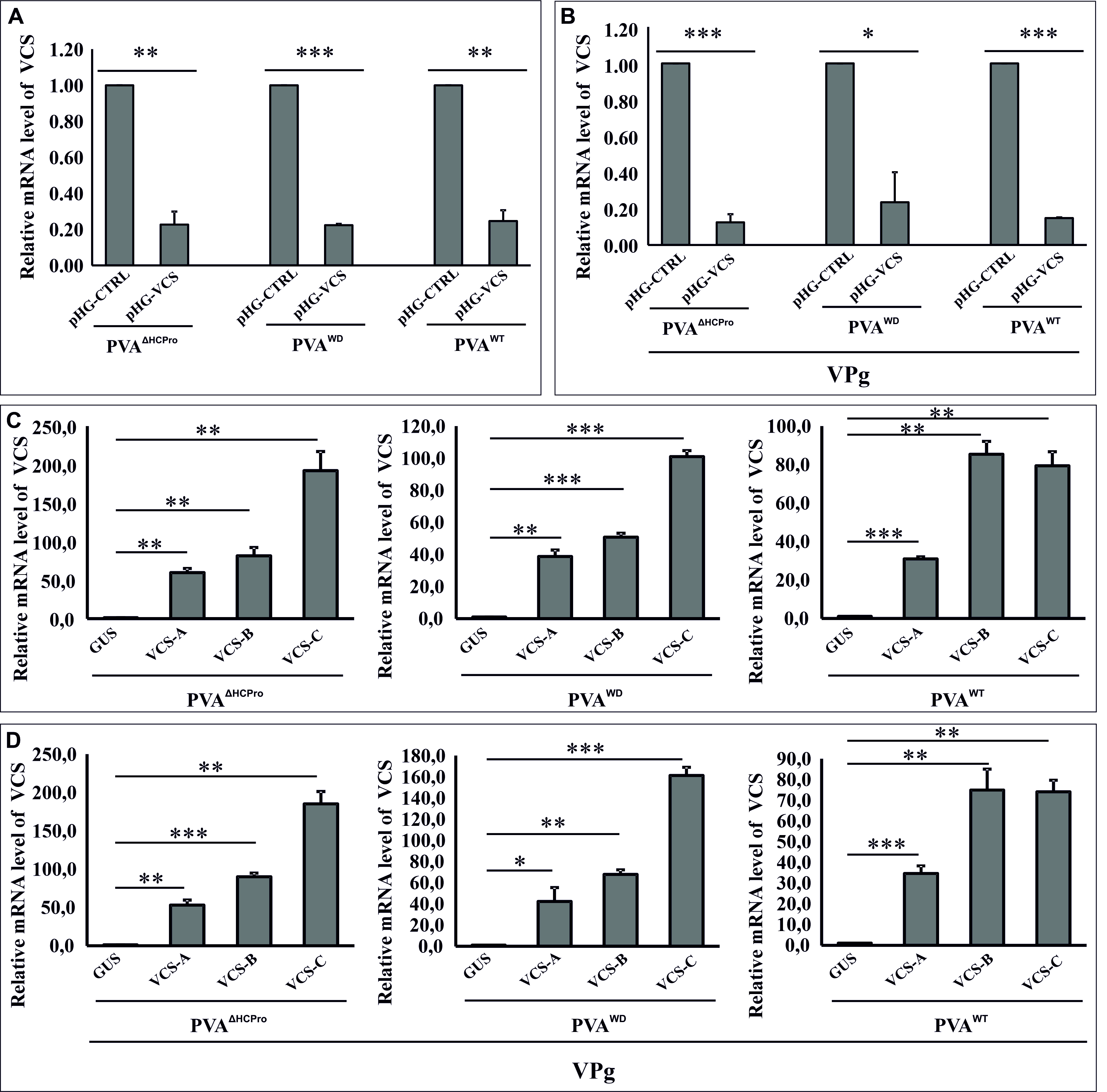

Supplement: S13 Fig — (A, B) For validation of VCS silencing in Fig 7B and 7C, VCS mRNA levels were detected by qPCR using primer pair recognizing all three forms of VCS. (C, D) For confirmation of VCS overexpression in Fig 7D and 7E, qPCRs were carried out with primers specific to VCS-A, VCS-B and VCS-C respectively. Significance of the differences between the compared samples is denoted by asterisk (*P < 0.05, **P < 0.01, ***P < 0.001). (TIF) [file ppat.1008956.s013.tif]

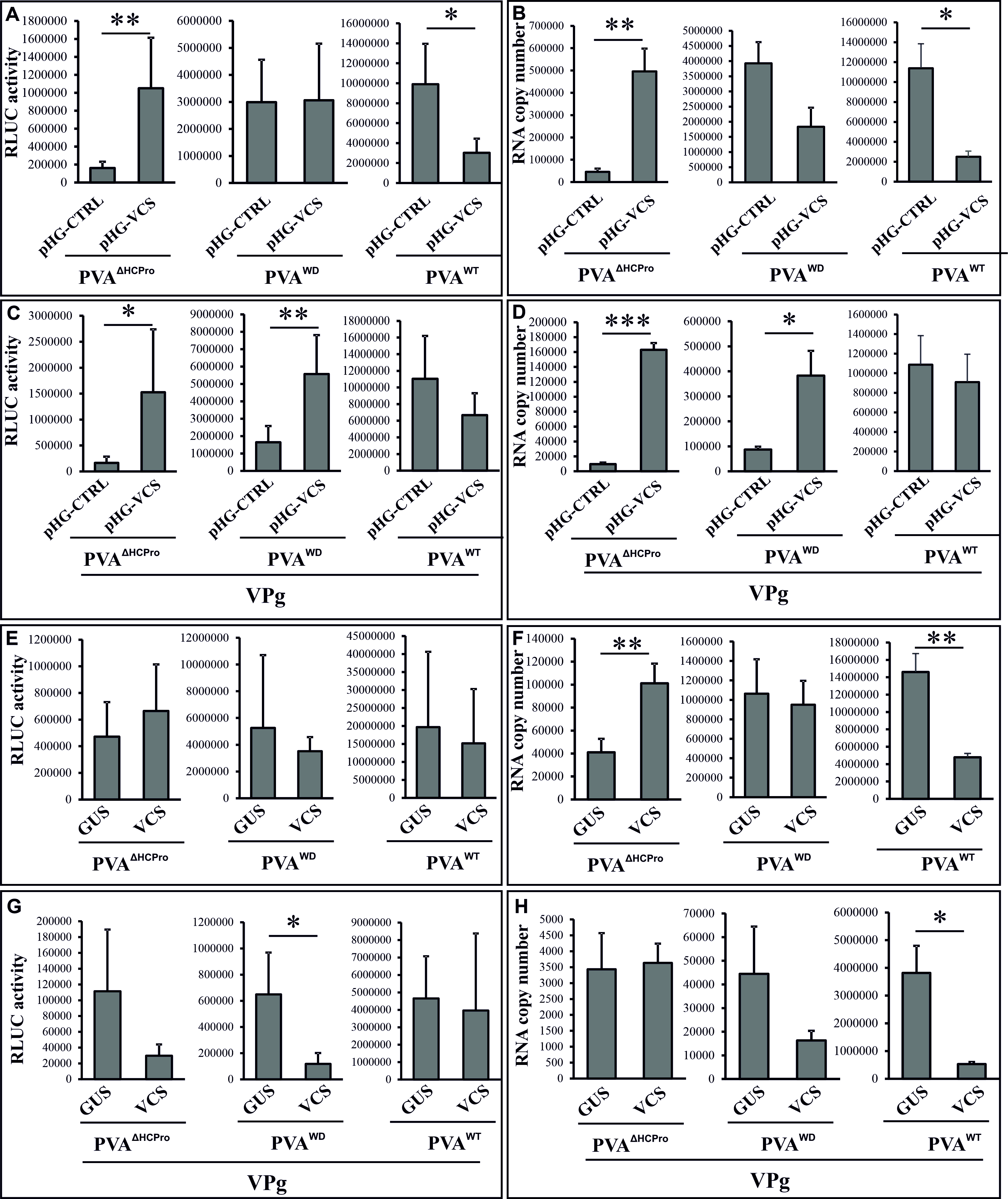

Supplement: S14 Fig — (A) Amount of RLUC activity detected from replicating variants of PVA- PVAΔHCPro, PVAWD and PVAWT upon VCS-silencing (B) Amount of RNA accumulation detected from replicating variants of PVA- PVAΔHCPro, PVAWD and PVAWT upon VCS-silencing (C) Amount of RLUC activity detected from replicating variants of PVA- PVAΔHCPro, PVAWD and PVAWT upon VCS-silencing and VPg overexpression (D) Amount of RNA accumulation detected from replicating variants of PVA- PVAΔHCPro, PVAWD and PVAWT upon VCS-silencing and VPg overexpression (E) Amount of RLUC activity detected from replicating variants of PVA- PVAΔHCPro, PVAWD and PVAWT upon VCS overexpression (F) Amount of RNA accumulation detected from replicating variants of PVA- PVAΔHCPro, PVAWD and PVAWT upon VCS overexpression (G) Amount of RLUC activity detected from replicating variants of PVA- PVAΔHCPro, PVAWD and PVAWT upon VCS and VPg overexpression (H) Amount of RNA accumulation detected from replicating variants of PVA- PVAΔHCPro, PVAWD and PVAWT upon VCS and VPg overexpression. Significance of the differences between the compared samples is denoted by asterisk (*P < 0.05, **P < 0.01). (TIF) [file ppat.1008956.s014.tif]

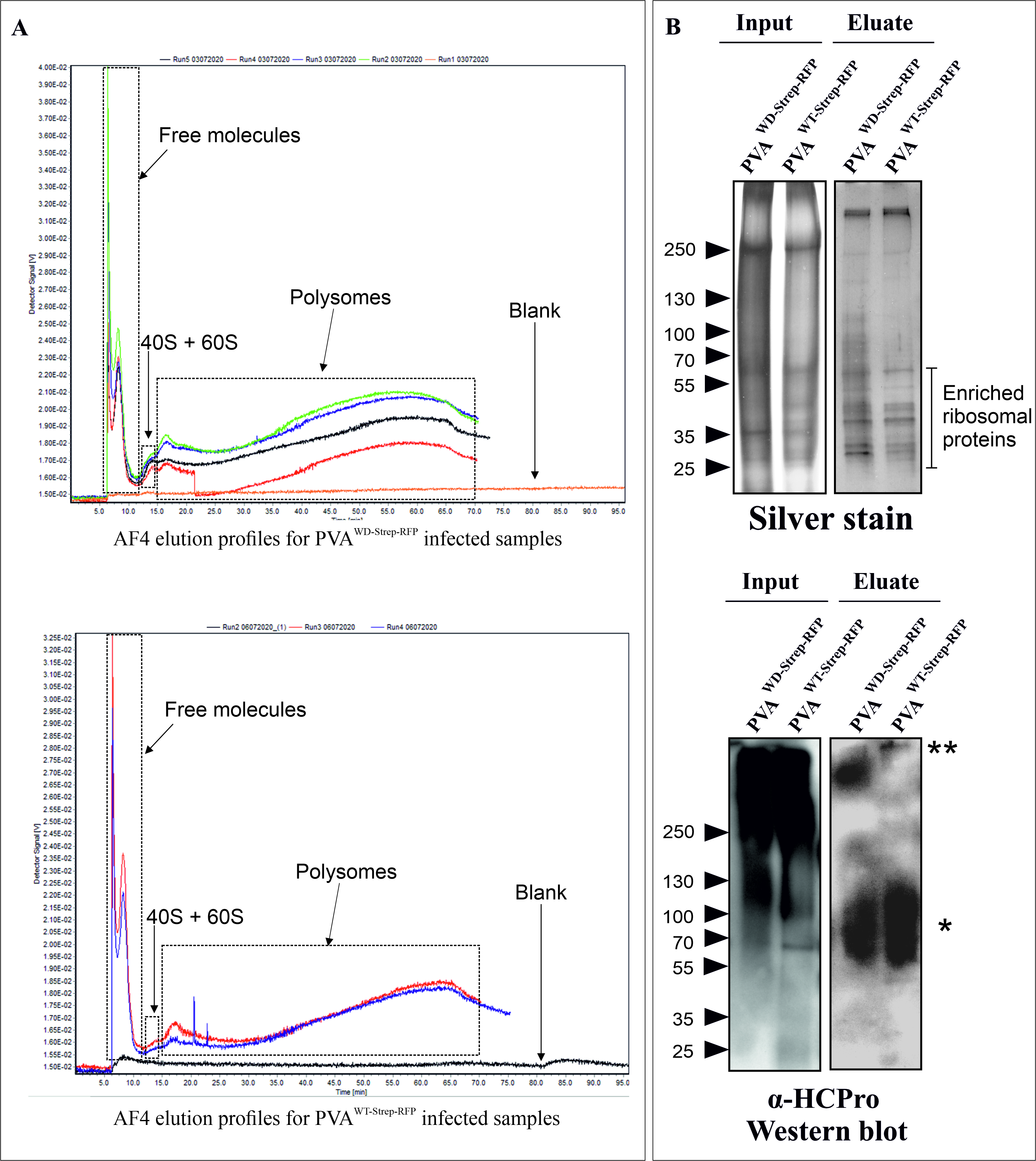

Supplement: S15 Fig — Polysomes were purified from PVAWD-Strep-RFP (OD600 = 0.3) and PVAWT-Strep-RFP (OD600 = 0.1) infected N. benthamiana plants (sampled at 4 dpi), via a Assymetric Field Flow Fractionation (AF4) protocol previously described in Eskelin et al. (2019). (A) Upper panel presents AF4 elution profiles of representative PVAWD-Strep-RFP infected samples while the lower panel presents those of representative PVAWT-Strep-RFP infected samples. Polysome fraction from the eluate (between 15–70 min) were pooled together and concentrated using a 10kDa MW cutoff centrifugation filter (Amicon). (B) Concentrated eluates were subjected to SDS-PAGE and the gels were silver stained (upper panel) and subjected to western blotting with anti-HCPro antibody (lower panel). Silver stained gel images for concentrated eluates show clear enrichment of ribosomal proteins (marked in the image between 55-25 kDa). The western blot revealed signals corresponding to both HCProWD and HCProWT both in the monomeric (marked by ‘*’) and HMW region (marked by ‘**’). Interestingly, band for HCProWD-Strep-RFP at the HMW region was visibly lower than that of the HCProWT-Strep-RFP. (TIF) [file ppat.1008956.s015.tif]

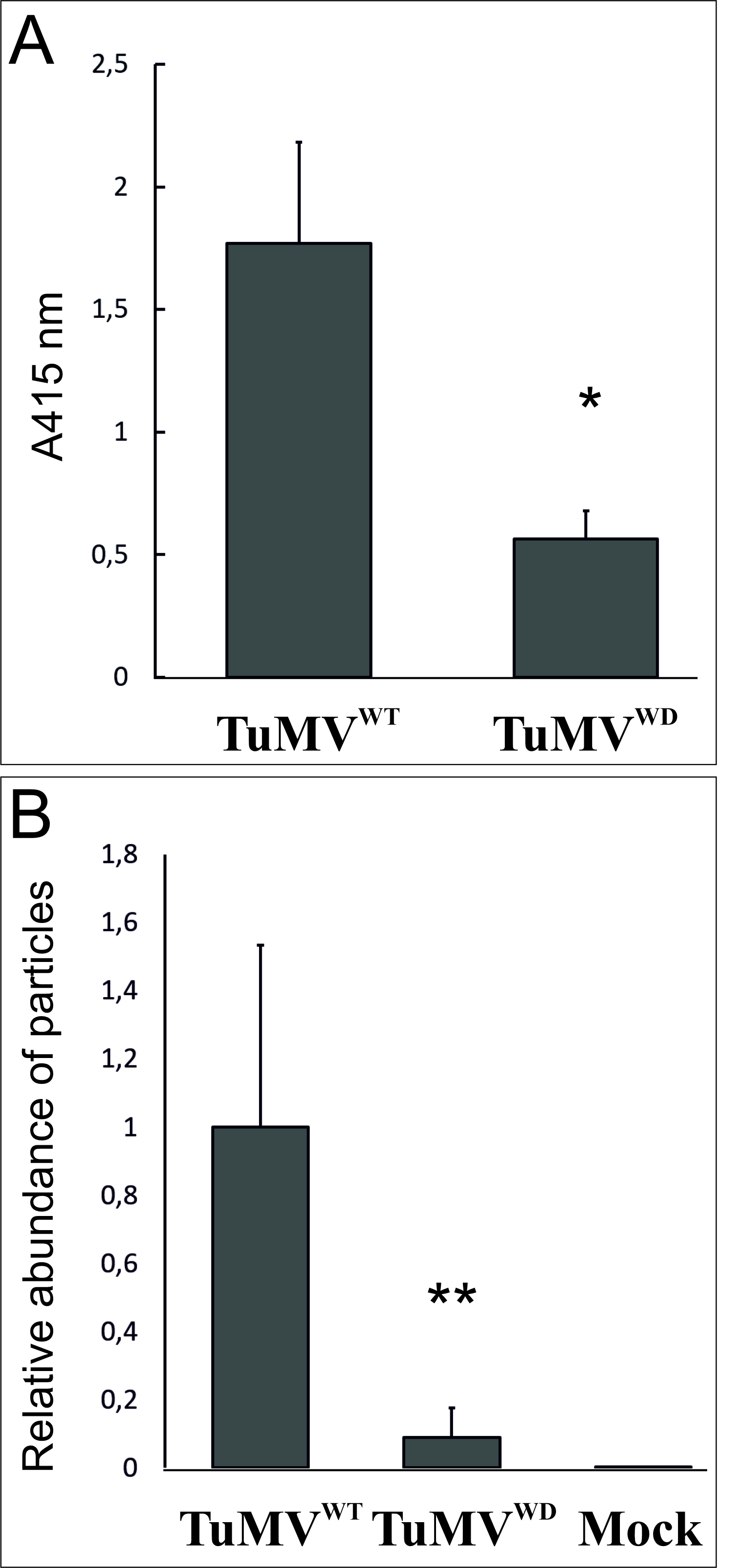

Supplement: S16 Fig — Systemic infection and particle abundance are reduced in TuMVWD compared to the control virus TuMVWT. Student’s t-test was used to calculate satistical significance (Significance of the differences between the compared samples is denoted by asterisk (*P < 0.05, **P < 0.01). (A) Abundance of TuMV CP in systemically infected N. benthamiana at 14 dpi. Plants were infiltrated by TuMVWT and TuMVWD (OD600 = 0.5) and samples from systemic leaves were analysed by anti-TuMV CP ELISA (Agdia). (B) Relative TuMV particle abundance in systemically infected N. benthamiana leaves at 12 dpi (OD600 = 0.5). Particle abundance was measured by IC-RT-PCR. (TIF) [file ppat.1008956.s016.tif]

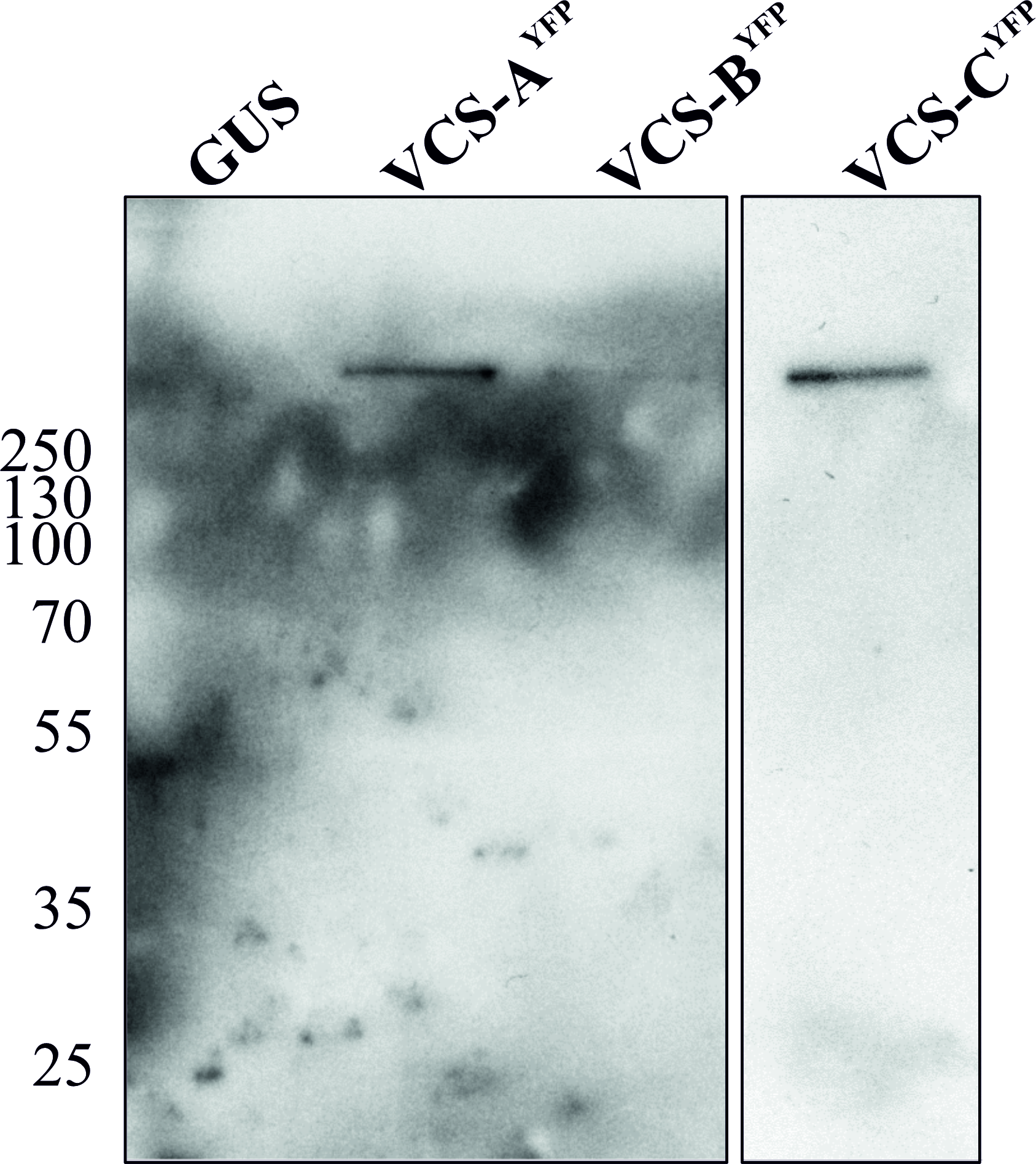

Supplement: S17 Fig — α-VCS western blot showing that all three forms of VCS are recognized by the anti-VCS antibody. For this experiment VCS-AYFP, VCS-BYFP and VCS-CYFP were overexpressed independently in N. benthamiana (infiltrated at OD600 0.5). Samples were collected at 3 dpi followed by affinity purification by GFP trap (ChromoTek). The eluates were analyzed by SDS-PAGE and anti-VCS western blot (antibody dilution 1 μg/ml). (TIF) [file ppat.1008956.s017.tif]
