# Supplementary material for: Association of host protein VARICOSE with HCPro within a multiprotein complex is crucial for RNA silencing suppression, translation, encapsidation and systemic spread of potato virus A infection
Source: PLoS Pathog. 2020 Oct 12;16(10):e1008956. doi: 10.1371/journal.ppat.1008956 (PMC7581364; doi:10.1371/journal.ppat.1008956)
Supplement: S1 Table — (DOCX) [file ppat.1008956.s018.docx]

**S1 Table** Recombinant constructs used in this study.

| **Construct name** | **Gene Cassette** | **Vector** | **Description** | **References** |
| --- | --- | --- | --- | --- |
| **PVA^WT^** | 35S-PVA^WT^:RLUC^int^-nos | pRD400 | RLUC-tagged full-length infectious cDNA clone of PVA | Eskelin et al., 2010 |
| **PVA^WD^** | 35S-PVA^WD^:RLUC^int^-nos | pRD400 | RLUC-tagged full-length infectious cDNA clone of PVA with mutation in WD domain interacting motif of HCPro | This study |
| **PVA^ΔGDD-HCProWT^** | 35S-PVA-[HCPro^WT^- NIb^ΔGDD^] ::RLUC^int^-nos | pRD400 | RLUC-tagged non-replicating variant of PVA with mutation in GDD motif of its replicase. HCPro expressed is wild type | Hafrén et al., 2015 |
| **PVA^ΔGDD-HCProWD^** | 35S-PVA-[HCPro^WD^- NIb^ΔGDD^] ::RLUC^int^-nos | pRD400 | RLUC-tagged non-replicating variant of PVA with mutation in GDD motif of its replicase. HCPro expressed has mutation in its WD domain interacting motif | This study |
| **PVA^WT-Strep-RFP^** | 35S-PVA-[(2xStrep)-RFP-HCPro^WT^]::RLUC^int^-nos | pRD400 | RLUC-tagged PVA expressing HCPro^WT^ fused to the red fluorescent protein (RFP) and two copies of the Strep-tag II | Hafrén et al., 2015 |
| **PVA^WD-Strep-RFP^** | 35S-PVA-[(2xStrep)-RFP-HCPro^WD^]::RLUC^int^-nos | pRD400 | RLUC-tagged PVA expressing HCPro^WD^ fused to the red fluorescent protein (RFP) and two copies of the Strep-tag II | This study |
| **HCPro^WT^** | 35S-[(2xStrep)-RFP-HCPro^WT^]-nos | pRD400 | Plasmid expressing PVA HCPro^WT^ | Hafrén et al., 2015 |
| **HCPro^WD^** | 35S-HCPro^WD^-nos | pRD400 | Plasmid expressing PVA HCPro^WD^ | This study |
| **HCPro^WT-Strep-RFP^** | 35S-[(2xStrep)-RFP-HCPro^WT^]-nos | pRD400 | Plasmid expressing PVA HCPro^WT^ tagged Two copies of Strep-tag II and RFP | This study |
| **HCPro^WD-Strep-RFP^** | 35S-[(2xStrep)-RFP-HCPro^WD^]-nos | pRD400 | Plasmid expressing PVA HCPro^WD^ tagged Two copies of Strep-tag II and RFP | This study |
| **PVA^ΔHCPro^** | 35S-PVA^ΔHCPro^::RLUC-nos | pRD400 | PVA tagged with RLUC lacking HCPro | Hafrén et al., 2015 |
| **PVA^ΔGDD-ΔHCPro^** | 35S-PVA^ΔHCPro^::RLUC-nos | pRD400 | RLUC-tagged non-replicating variant of PVA with a mutation in GDD motif of its replicase. This PVA construct lacks HCPro | Hafrén et al., 2015 |
| **pHG-CTRL** | 35S-(empty hp)-ocs | pHG12 | Plasmid expressing no hairpin RNA | Hafrén et al., 2013 |
| **pHG-VCS** | 35S-VCS(hp)-ocs | pHG12 | Plasmid expressing hairpin RNA targeting the VCS gene family | Hafrén et al., 2015 |
| **pHG-RLUC** | 35S-RLUC(hp)-ocs | pHG12 | Plasmid expressing hairpin RNA targeting the RLUC | Hafrén et al., 2015 |
| **VCS-A** | 35S-VCS-A-nos | pRD400 | Plasmid expressing *N. benthamiana* VCS-A | This study |
| **VCS-B** | 35S-VCS-B-nos | pRD400 | Plasmid expressing *N. benthamiana* VCS-B | This study |
| **VCS-C** | 35S-VCS-C-nos | pRD400 | Plasmid expressing *N. benthamiana* VCS-C | This study |
| **VCS-A^YFP^** | 35S-VCS-A^YFP^-nos | pRD400 | Plasmid expressing *N. benthamiana* VCS-A tagged with YFP | This study |
| **VCS-B^YFP^** | 35S-VCS-B^YFP^-nos | pRD400 | Plasmid expressing *N. benthamiana* VCS-B tagged with YFP | This study |
| **VCS-C^YFP^** | 35S-VCS-C^YFP^-nos | pRD400 | Plasmid expressing *N. benthamiana* VCS-C tagged with YFP | This study |
| **HCPro^SDM^** | 35S-HCPro^SDM-RFP^-nos | pSITEII-6C1 | Plasmid expressing silencing suppression deficient mutant of HCPro fused to RFP | Hafrén et al., 2015 |
| **HCPro^4EBM^** | 35S-HCPro^4EBM-RFP^-nos | pSITEII-6C1 | Plasmid expressing eIF4E binding deficient mutant of HCPro fused to RFP | Hafrén et al., 2015 |
| **VPg** | 35S-VPg-nos | pRD400 | Plasmid expressing PVA VPg | Eskelin et al., 2011 |
| **P0^YFP^** | 35S-P0^YFP^-nos | pRD400 | Plasmid expressing *N. benthamiana* P0 tagged with YFP | Hafrén et al., 2015 |
| **P0^CFP^** | 35S-P0^YFP^-nos | pRD400 | Plasmid expressing *N. benthamiana* P0 tagged with CFP | This study |
| **GUS** | 35S-GUS-nos | pRD400 | Plasmid expressing uidA gene encoding β-glucuronidase (GUS) | Eskelin et al., 2010 |
| **FLUC** | 35S-FLUC-nos | pRD400 | Plasmid expressing intron-spliced FLUC | Eskelin et al., 2010 |
