# Supplementary material for: Association of host protein VARICOSE with HCPro within a multiprotein complex is crucial for RNA silencing suppression, translation, encapsidation and systemic spread of potato virus A infection
Source: PLoS Pathog. 2020 Oct 12;16(10):e1008956. doi: 10.1371/journal.ppat.1008956 (PMC7581364; doi:10.1371/journal.ppat.1008956)
Supplement: S2 Table — (DOCX) [file ppat.1008956.s019.docx]

**Table S2** LC-MS/MS identification of proteins present in HMW complexes produced by HCPro^WT-Strep-RFP^ but not in HCPro^WD-Strep-RFP^

| Protein | Mock Purification | HCPro^WD-Strep-RFP^ Purification (mean PSM* values) | HCPro^WT-Strep-RFP^ Purification (mean PSM* values) | Western blot validation | Remarks |
| --- | --- | --- | --- | --- | --- |
| SAMS | 0 | 0 | 2.66 | Yes | Reduced in HCPro^WD-Strep-RFP^ |
| CI | 0 | 0 | 1 | Yes | Reduced in HCPro^WD-Strep-RFP^ |
| eIF4A | 0 | 0 | 1 | No |  |
| VPg | 0 | 0 | 0.66 | Yes | Reduced in HCPro^WD-Strep-RFP^ |

* Mean PSM values are calculated from the individual PSM values obtained from three biological replicates
