# Supplementary material for: Association of host protein VARICOSE with HCPro within a multiprotein complex is crucial for RNA silencing suppression, translation, encapsidation and systemic spread of potato virus A infection
Source: PLoS Pathog. 2020 Oct 12;16(10):e1008956. doi: 10.1371/journal.ppat.1008956 (PMC7581364; doi:10.1371/journal.ppat.1008956)
Supplement: S1 Reference — (DOCX) [file ppat.1008956.s021.docx]

**S1 References:**

**Eskelin, K., Hafrén, A., Rantalainen, K.I. and Mäkinen, K.** (2011) Potyviral VPg enhances viral RNA Translation and inhibits reporter mRNA translation in planta. *J Virol*, **85**, 9210-9221.

**Eskelin, K., Suntio, T., Hyvärinen, S., Hafrén, A. and Mäkinen, K.** (2010) Renilla luciferase-based quantitation of Potato virus A infection initiated with Agrobacterium infiltration of N. benthamiana leaves. *Journal of Virological Methods*, **164**, 101-110.

**Hafrén, A., Eskelin, K. and Mäkinen, K.** (2013) Ribosomal protein P0 promotes Potato virus A infection and functions in viral translation together with VPg and eIF(iso)4E. *J Virol*, **87**, 4302-4312.

**Hafrén, A., Lõhmus, A. and Mäkinen, K.** (2015) Formation of Potato Virus A-Induced RNA Granules and Viral Translation Are Interrelated Processes Required for Optimal Virus Accumulation. *PLoS Pathog*, **11**, e1005314.

**Eskelin, K., Varjosalo, M., Ravantti, J. and Mäkinen, K.** (2019) Ribosome profiles and riboproteomes of healthy and Potato virus A- and Agrobacterium-infected Nicotiana benthamiana plants. *Mol Plant Pathol.*, **20**, 392-409.
